# Beyond Inflammation: The Role of Oxidative Stress and Gut–Skin Axis Dysbiosis in the Pathogenesis of Immune-Mediated Skin Disorders and Potential Therapeutic Implications

**DOI:** 10.3390/ijms27114656

**Published:** 2026-05-22

**Authors:** Maria Clara Gama de Souza Silva, Lucrezia De Pietro, Carla Ruffino San Cataldo, Antonio Bisaccia, Federica Nuccio, Federica Li Pomi, Sebastiano Gangemi

**Affiliations:** 1 School and Operative Unit of Allergy and Clinical Immunology, Department of Clinical and Experimental Medicine, University of Messina, 98125 Messina, Italy; claragamasouza@gmail.com (M.C.G.d.S.S.); lucreziadepietro@gmail.com (L.D.P.); carlaruffino1@gmail.com (C.R.S.C.); antoniobisaccia01@gmail.com (A.B.); federica.nuccio01@gmail.com (F.N.); sebastiano.gangemi@unime.it (S.G.); 2 Department of Precision Medicine in Medical, Surgical and Critical Care (Me.Pre.C.C.), University of Palermo, 90127 Palermo, Italy

**Keywords:** oxidative stress, lipid peroxidation, ferroptosis, reactive oxygen species, microbiome, skin diseases, inflammation, dysbiosis

## Abstract

The skin is a complex immunological organ in which reactive oxygen species (ROS)-related pathways and host–microbe interactions synergically maintain immune homeostasis. Dysregulation of several oxidative mechanisms, including lipid peroxidation, mitochondrial dysfunction, ferroptosis, and impaired antioxidant defenses, alongside gut microbiome imbalance, is increasingly recognized as a key modulator of the immune response involved in disease onset and progression. However, their role in immune-mediated dermatoses remains incompletely defined. This narrative review aims to provide a comprehensive overview of the contribution of these altered pathways to the pathogenesis and prognosis of the major immune-mediated skin diseases. Across all conditions examined, elevated oxidative biomarkers, such as malondialdehyde (MDA), advanced glycation end-products (AGEs), advanced oxidation protein products (AOPPs), 8-hydroxydeoxyguanosine (8-OHdG), and reduced antioxidant capacity are consistently reported. Ferroptosis, driven by iron-dependent lipid peroxidation and dysfunction of Glutathione peroxidase 4 (GPX4), emerges as a relevant cell death pathway, particularly in psoriasis and atopic dermatitis (AD). In parallel, dysbiosis of the gut and skin microbiomes, characterized by depletion of short-chain fatty acid (SCFA)-producing taxa such as *Faecalibacterium prausnitzii*, *Bifidobacterium*, and *Akkermansia muciniphila*, has been reported across multiple diseases. Particular attention is given to shared molecular axes, such as the disruption of epithelial barrier integrity, activation of innate and adaptive immune responses, and the role of microbial-derived metabolites in modulating redox signaling, unraveling a bidirectional crosstalk. Emerging therapeutic strategies targeting these bidirectional crosstalks show biological plausibility and promising preliminary results. Integrating redox and microbial profiling into clinical practice may improve patient stratification and foster the development of more personalized therapeutic approaches beyond conventional immunological treatments.

## 1. Background

### 1.1. Oxidative Stress in Skin Immunopathology

Redox regulation is a fundamental biological process that allows cells to adapt to metabolic, environmental, and immunological stimuli. In this context, reactive oxygen species (ROS) are not merely harmful by-products of aerobic metabolism, but essential signaling molecules involved in immune defense, cellular adaptation, keratinocyte function, and tissue repair [1]. Oxidative stress (OS) occurs when ROS generation exceeds the capacity of antioxidant and repair systems, leading to disruption of cellular redox homeostasis. This imbalance may result in lipid peroxidation, protein oxidation, DNA damage, mitochondrial dysfunction, and activation of inflammatory pathways [1,2]. The distinction between physiological and pathological redox signaling is particularly relevant in the skin. Controlled ROS production, referred to as oxidative eustress, supports barrier integrity and immune homeostasis, whereas excessive or persistent ROS accumulation induces oxidative distress and tissue injury [2]. Because the skin is continuously exposed to environmental oxidants, microbial stimuli, ultraviolet radiation, and endogenous metabolic ROS, cutaneous redox homeostasis represents a dynamic equilibrium that is essential for maintaining barrier function and preventing chronic inflammation [1].

As the body’s largest organ, the skin functions as both a physical and biological barrier, protecting internal tissues from external insults such as air pollutants, ionizing and non-ionizing radiation, toxins, mechanical injury, and microbial invasion [1,3].

In this context, cutaneous antioxidant defense mechanisms neutralize excess oxidants and preserve redox balance, enabling adaptation to environmental fluctuations while protecting cellular integrity [4]. Consistently, moderate ROS levels participate in essential signaling pathways, whereas excessive accumulation leads to oxidative damage [1,5].

ROS can be classified as endogenous or exogenous based on their biological source, a distinction that helps clarify how physiological metabolism and environmental exposure jointly contribute to the cutaneous oxidative burden. Endogenous ROS are primarily generated as by-products of normal cellular metabolism and enzymatic reactions [6]. Key sources include the mitochondrial electron transport chain, xanthine oxidoreductase (XOR), nicotinamide adenine dinucleotide phosphate (NADP) oxidases, cytochrome P450 enzymes, peroxidases, cyclooxygenases, and lipoxygenases. Mitochondria and the endoplasmic reticulum are major contributors. Within mitochondria, molecular oxygen is converted to superoxide anion (O_2_•^−^) by complexes I and III or xanthine oxidase (XO), then dismutated to hydrogen peroxide (H_2_O_2_) by superoxide dismutase (SOD). Under normal conditions, H_2_O_2_ is detoxified into water and oxygen by antioxidant enzymes, including thioredoxin peroxidases (TPx), glutathione peroxidases (GPxs), and catalase (CAT). Under OS, H_2_O_2_ can participate in Fenton reactions, thereby generating additional ROS. Peroxisomal β-oxidation of fatty acids also produces H_2_O_2_, and nitric oxide (NO•) is synthesized during protein production via nitric oxide synthases [6].

Exogenous ROS are predominantly induced by environmental factors, with ultraviolet (UV) radiation being one of the most significant sources in the skin. UVA penetrates the dermis, contributing to tanning, whereas UVB affects the epidermis, causing sunburn. Both UVA and UVB stimulate ROS formation, contributing to OS, cellular damage, pigmentation, and photoaging [6,7]. Photosensitizing molecules such as psoralens, porphyrins, and tetracyclines can convert molecular oxygen to singlet oxygen (^1^O_2_), a potent oxidizing agent with a very short half-life (10^−6^–10^−5^ s). ^1^O_2_ can rapidly initiate secondary oxidative reactions leading to the generation of ROS such as O_2_•^−^, H_2_O_2_, and •OH [5].

ROS may also arise enzymatically. XO generates superoxide anions in keratinocytes and vascular endothelial cells, with activity increased by ischemia–reperfusion or light irradiation. NO•, produced by neuronal, inducible, and endothelial NOS isoforms, regulates epidermal proliferation, keratinization, and dermal blood flow, while iNOS contributes to inflammatory ROS production in conditions such as psoriasis and UV-induced damage [5].

ROS are normally produced in aerobic metabolism and participate in essential cellular processes [8]. Excess ROS, however, damages lipids, proteins, carbohydrates, and DNA, causing membrane disruption, molecular fragmentation, macromolecule cross-linking, and potentially apoptosis or necrosis [8]. ROS also mediate signaling pathways such as MAPK, JAK/STAT, PI3K/AKT/mTOR, NF-κB, Nrf2, and SIRT1/FOXO, altering cytokine production and enzymatic expression, and thereby further affecting cellular function [6].

Excess ROS and reactive nitrogen species (RNS) from UV exposure are closely associated with skin pigmentation and aging, influencing melanogenesis signaling and contributing to oxidation or polymerization of melanin and its precursors. Antioxidants counteract these effects by scavenging reactive species, inhibiting melanogenic signaling, and protecting melanocytes [7].

Direct measurement of ROS in skin diseases is difficult, but evidence indicates that ROS contribute to the pathogenesis of various dermatological conditions, including inflammatory, autoimmune, and degenerative disorders [5], finally leading to cellular dysfunction, inflammation, and in some cases carcinogenesis [6].

Thus, OS not only disrupts cellular homeostasis but also activates regulated cell death pathways, with potential implications for skin pathologies.

Excessive ROS have been shown to induce cellular and tissue damage in various ways, including the accumulation of AGEs. AGEs are heterogeneous products derived from proteins, lipids, and nucleic acids, and have been linked to a wide range of diseases as well as to skin aging. Their biological effects are largely mediated through interaction with the receptor for advanced glycation end products (RAGE), a multiligand receptor originally described by Neeper et al. in 1992. RAGE binds not only AGEs, its canonical ligands, but also several endogenous and exogenous molecules, including damage-associated molecular patterns (DAMPs) released by stress or dying cells [9]. Through RAGE activation, these ligands promote inflammation and cytokine production [10,11].

Given the central role of OS in skin physiology and pathology, mechanisms of redox-driven cell death, such as ferroptosis, have gained increasing attention. The skin, being continuously exposed to environmental oxidants and enriched in polyunsaturated lipids, may be particularly susceptible to lipid peroxidation-driven damage, positioning ferroptosis as a relevant pathway in cutaneous homeostasis and disease.

Ferroptosis is defined as an iron-dependent form of regulated cell death characterized by lipid peroxidation and driven by metabolic imbalance rather than caspase activation. This process can be conceptualized within a broader “redox network axis,” an integrated system comprising redox-active organelles—including mitochondria, peroxisomes, lysosomes, the endoplasmic reticulum, and the plasma membrane—together with transition-metal pools (labile Fe^2+^/Fe^3+^ and Cu^+^/Cu^2+^), low–molecular-weight redox couples, and oxidizable lipid substrates, particularly polyunsaturated fatty acid (PUFA)-containing phospholipids. These components collectively maintain local redox balance while supplying substrates for oxidative reactions [1,12,13].

Notably, microbial metabolites may also modulate ferroptosis susceptibility: SCFAs such as butyrate have been shown to regulate ferroptosis sensitivity through FFAR2–mTOR signaling and to suppress pro-inflammatory mediator synthesis, thereby indirectly influencing redox balance, lipid peroxidation, and iron metabolism [14].

Overall, ferroptosis represents a unique form of regulated cell death arising from the imbalance between iron-driven oxidative damage and cellular antioxidant defenses. Rather than being an isolated metabolic event, it emerges from the dynamic interplay between redox homeostasis, immune signaling, and environmental inputs. In this context, microbial metabolites further expand the regulatory landscape by modulating inflammation, redox tone, and cellular susceptibility to lipid peroxidation. This integrated perspective highlights ferroptosis as a multifactorial process positioned at the crossroads of metabolism, immunity, and host–microbiota interactions, underscoring its context-dependent role in both physiological regulation and disease pathogenesis.

### 1.2. Skin Microbiome and Homeostasis

The skin and mucosal membranes serve as primary host defenses by creating barriers against noxious external factors. This barrier is not merely structural, but results from the coordinated interaction of physical, chemical, immune, and microbial components [15,16]. The microorganisms present on the skin surface and mucous membranes—including opportunistic, pathogenic, and commensal species—are collectively referred to as the microbiota, whereas the term microbiome encompasses their collective genetic content and functional potential [17]. Within this ecosystem, microbial communities contribute actively to barrier function rather than representing passive colonizers of the skin surface. The skin microbiome is taxonomically diverse and varies across individuals, anatomical sites, and skin microenvironments. The epidermal microbiome is more directly exposed to environmental fluctuations, whereas deeper microbial communities appear relatively more stable and less influenced by external factors [15,16].

Upon exposure to pathogens, epithelial and immune cells detect microbial components through pattern recognition receptors (PRRs), including toll-like receptors (TLRs) and NOD-like receptors (NLRs), which recognize bacterial, viral, and fungal structures [18,19]. Activation of these receptors induces the release of pro-inflammatory cytokines such as IL-1 and IL-18, initiating immune responses. Thus, microbial sensing represents a critical interface through which environmental exposure is translated into barrier defense, cytokine production, and cutaneous immune activation.

The microbiota contributes to skin homeostasis through local and systemic mechanisms. At the local level, microbial metabolites support lipid organization, epithelial integrity, and immune tolerance. For instance, *Staphylococcus epidermidis* (*S. epidermidis*) produces sphingomyelinase, thereby increasing ceramide levels and enhancing barrier integrity [20]. Additionally, microbial-derived ligands activate the aryl hydrocarbon receptor (AHR) in keratinocytes, promoting differentiation and barrier repair [21]. Together, these mechanisms indicate that commensal microorganisms actively participate in epidermal remodeling, barrier maintenance, and regulation of cutaneous inflammation.

The development of the skin microbiome begins early in life and is influenced by maternal microbiota, delivery mode, and environmental exposure [22,23]. In adults, the microbiome varies by anatomical site: sebaceous areas are dominated by *Cutibacterium* species, moist regions by *Staphylococcus* and *Corynebacterium*, and dry areas show greater microbial diversity [24,25,26]. This site-specific distribution reflects differences in sebum content, humidity, pH, and immune microenvironment, all of which shape microbial composition and function.

Alterations in microbiome composition, defined as dysbiosis, are associated with several dermatological conditions. In psoriasis, an increased abundance of *Proteobacteria*, along with shifts in *Streptococcus* and *Cutibacterium* species, has been reported [24,25]. Similar dysbiotic patterns have been observed in atopic dermatitis and acne, in which disease is often linked to specific pathogenic strains rather than to overall bacterial presence [26]. These observations suggest that disease-associated dysbiosis involves not only taxonomic shifts, but also functional changes in microbial metabolism, barrier regulation, and host–microbe immune interactions.

Despite the critical role of the skin microbiome in maintaining local homeostasis, increasing evidence indicates that the gut microbiome also contributes to the development and progression of dermatological diseases. As the largest and most metabolically active microbial reservoir of the human body, the gut microbiome exerts systemic immunological and metabolic effects that may influence distant organs, including the skin [27,28].

The formation of the gut microbiota begins early in life and is influenced by both prenatal and postnatal factors. Evidence suggests that microbial exposure may occur during fetal development, as microbial signatures have been detected in meconium and oral samples of neonates, including preterm infants [29].

Following birth, the intestinal microbiota undergoes progressive colonization, reaching an adult-like composition between approximately 2 and 5 years of age [30]. This early-life maturation is relevant because microbial exposures during this window may shape immune education and later susceptibility to inflammatory or immune-mediated diseases.

In this context, circulating microbial metabolites may reach peripheral tissues, including the skin, where they can influence barrier integrity and immune responses [31].

Within this framework, the gut microbiota primarily exerts systemic effects through metabolic and immune signaling, whereas the skin microbiota predominantly regulates local barrier integrity and cutaneous immune responses. The interaction between these two microbial ecosystems is therefore conceptualized as the gut–skin axis (GSA), a bidirectional communication network linking intestinal and cutaneous homeostasis through immune, endocrine, metabolic, and neural pathways [32,33]. This bidirectional communication is further integrated into the brain–gut-skin axis (BGSA), which incorporates the central nervous system into the gut-skin framework [12,13,34]. In this broader model, psychological stress, neuroendocrine mediators, immune activation, and microbial-derived signals converge to regulate inflammation and tissue homeostasis. Stress-induced neuroimmune responses contribute to the exacerbation of skin inflammation and may further drive chronic disease activity through feedback mechanisms [15,16,17]. The concept of a gut–skin connection dates back to early observations linking emotional states and gastrointestinal function to skin disease [19]. However, modern research demonstrates that these interactions involve sophisticated neurocircuitry, neurotransmitter signaling, and immune modulation rather than simple hypothalamic–pituitary–adrenal (HPA) axis activation alone [20].

Microbial metabolites play a central role in mediating these systemic effects. SCFAs, tryptophan metabolites, and trimethylamine N-oxide (TMAO) influence immune regulation and skin physiology [32]. SCFAs, particularly butyrate, promote regulatory immune responses by inhibiting histone deacetylases and suppressing Nuclear factor kappa-light-chain-enhancer of activated B cells (NF-κB) signaling, thereby supporting epithelial integrity, wound healing, and immune tolerance [32]. Conversely, dysbiosis may lead to the production of harmful metabolites such as phenol and p-cresol, which impair epidermal differentiation and barrier function [33]. Therefore, the biological impact of the microbiome depends not only on microbial composition but also on the balance between protective and potentially harmful metabolic outputs.

In addition to SCFAs, the gut microbiome contributes to the production, transformation, or modulation of several bioactive molecules, including secondary bile acids, tryptophan-derived metabolites, GABA-related pathways, serotonin-related pathways, and dopamine-related signaling [34].

Secondary bile acids generated through microbial transformation of primary bile acids can enter the systemic circulation and interact with host receptors, such as the farnesoid X receptor-α (FXR), thereby contributing to the regulation of glucose homeostasis [35].

In addition to bile acid metabolism, gut microorganisms convert dietary choline into trimethylamine (TMA), a process involved in host lipid metabolism [36]. Collectively, these pathways may indicate that gut-derived metabolites may influence cutaneous inflammation indirectly by modulating systemic metabolism, immune polarization, and epithelial barrier responses.

Therapeutic modulation of the microbiome represents a promising strategy. Oral supplementation with *Lactobacillus* strains has been shown to improve skin barrier recovery and reduce inflammation, including mast cell activation, vasodilation, and cytokine release. Specific strains, such as *Lactobacillus paracasei* CNCM I-2116 and *Lactobacillus johnsonii*, demonstrate systemic immunomodulatory effects that support skin homeostasis. These findings may suggest that microbiome-directed approaches may complement conventional anti-inflammatory treatments, although their clinical efficacy is likely to depend on disease phenotype, microbial baseline composition, and strain-specific effects.

Overall, the microbiome plays a crucial role in maintaining skin barrier integrity and systemic homeostasis. However, further research is required to clarify the long-term impact of microbiome-targeted therapies and the contributions of non-bacterial components, including fungi and viruses, in dermatological diseases [21,33].

## 2. Aim of the Study

The purpose of this narrative review is to examine and integrate current knowledge on the role of OS pathways and microbiome dysregulation in the pathogenesis of major immune-mediated skin diseases and immunological interest. This review seeks to delineate the contribution of key oxidative mechanisms, including redox imbalance, lipid peroxidation, and ferroptosis; to characterize the principal alterations of the skin and gut microbiome; and to explore the bidirectional interplay between redox homeostasis and microbial composition in driving immune dysregulation. Moreover, we emphasize the shared pathogenic pathways across different dermatoses and discuss the potential clinical implications of targeting these interconnected mechanisms.

## 3. Role of Oxidative Stress and Microbiome in Skin Diseases

### 3.1. Psoriasis

Psoriasis is a chronic immune-mediated inflammatory skin disease affecting more than 100 million people worldwide and clinically characterized by erythematous, scaly plaques mainly involving the elbows, knees, trunk, and scalp [37]. Although traditionally defined by its cutaneous manifestations, psoriasis is now recognized as a systemic immune-mediated inflammatory disease frequently associated with comorbidities, including psoriatic arthritis, inflammatory bowel disease, cardiovascular disorders, metabolic syndrome, and depression [37]. Its pathogenesis is driven by complex interactions among keratinocytes, infiltrating immune cells, and resident skin cells, resulting in persistent inflammation, excessive keratinocyte proliferation, and abnormal epidermal differentiation [38,39,40].

**Figure 1:**
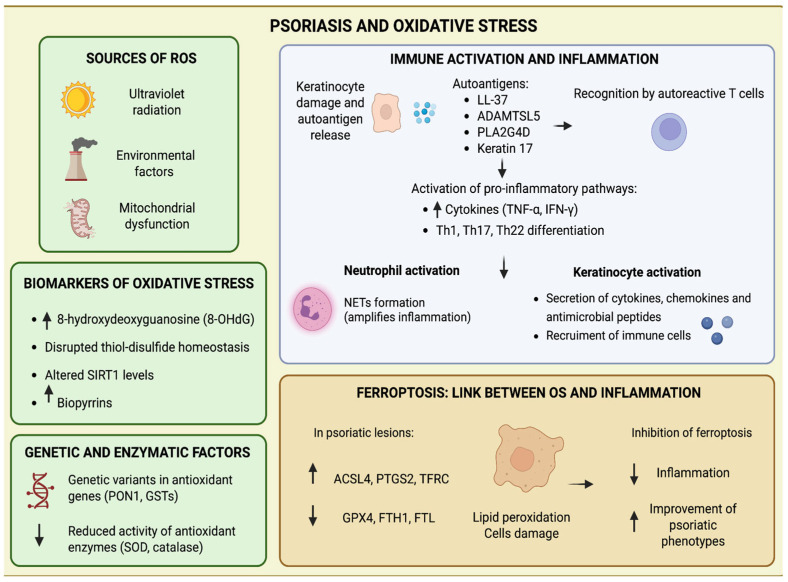
An overview of the main mechanisms related to oxidative stress involved in the pathogenesis of Psoriasis. Symbols: ↑, increased; ↓ reduced. Created in BioRender. Gama de Souza Silva, M. C. (2026) https://BioRender.com/zugnu6a (accessed on 13 May 2026).

Experimental and clinical evidence support the role of oxidative stress in psoriasis (see Table S1) (Figure 1). Within the psoriatic inflammatory microenvironment, ROS contribute to structural damage of the epidermis, particularly the stratum corneum, and promote immune activation [39,40,41,42]. This is supported by experimental models in which extracellular SOD deficiency enhances IL-23-mediated skin inflammation, as well as by clinical observations showing reduced α-tocopherol levels in patients with psoriasis [40,41]. External triggers, including ultraviolet radiation, may further increase ROS production and amplify cutaneous oxidative burden [39].

OS promotes the release of autoantigens from keratinocytes, including LL-37, ADAMTSL5, PLA2G4D, and keratin 17, which may be recognized by autoreactive T cells and stimulate pro-inflammatory cytokines such as TNF-α and IFN-γ, driving Th1, Th17, and Th22 differentiation [39,43]. This immune activation is further amplified by innate inflammatory mechanisms. Neutrophils amplify inflammation through the formation of neutrophil extracellular traps (NETs), while keratinocytes secrete cytokines, chemokines, and antimicrobial peptides, further recruiting immune cells [44,45].

Several biomarkers indicate OS in psoriasis, including elevated 8-OHdG, disrupted thiol–disulfide homeostasis, altered Sirtuin 1 (SIRT1) levels, and increased biopyrrins [46,47,48]. In addition to these oxidative biomarkers, ferroptosis has emerged as a novel mechanism linking OS and inflammation, with psoriatic lesions showing increased expression of ACSL4, PTGS2, and TFRC, and decreased expression of GPX4, FTH1, and FTL [49,50,51]. Inhibition of ferroptosis seems to reduce inflammation and improve psoriatic phenotypes [49].

Genetic variants in antioxidant genes (e.g., PON1, GSTs) and reduced activity of SOD and catalase are believed to contribute to OS susceptibility [32,52,53].

In addition to OS, increasing evidence supports a possible role for the GSA in the pathogenesis of psoriasis (Table S2). Psoriatic dysbiosis is characterized by reduced microbial diversity, loss of SCFA-producing taxa such as *Faecalibacterium prausnitzii* and *Akkermansia muciniphila*, and enrichment of potentially pro-inflammatory taxa [42,43,44]. This microbial imbalance may disrupt intestinal barrier integrity, allowing translocation of microbial components like lipopolysaccharide (LPS) and bacterial DNA into systemic circulation, thereby activating cutaneous and systemic immune responses [45,46].

Microbial metabolites such as SCFAs and tryptophan-derived molecules are likely to influence immune pathways, promoting regulatory T cell (Treg) differentiation and suppressing Th17 responses via histone acetylation and AHR signaling [39,48]. In this context, microbial-derived signals may also act on innate immune compartments. Innate lymphoid cells (ILC3s) in both the gut and skin are modulated by microbial signals and contribute to IL-17 and IL-22 production, suggesting a possible link between gut dysbiosis and skin inflammation [49].

Therapeutic interventions targeting the microbiome, including probiotics, prebiotics, dietary modifications, and fecal microbiota transplantation, have shown potential benefits in both experimental and clinical settings, with evidence suggesting reductions in disease severity and inflammatory markers [37,38,50,51]. Modulation of the skin microbiome through phototherapy and systemic therapies may also influence microbial composition.

However, these findings remain preliminary and require careful interpretation. Despite growing evidence, further research is needed to elucidate causal mechanisms and translate findings into therapeutic strategies [32].

These findings support a mechanistic link between OS and dysbiosis of the GSA. ROS-driven inflammation and lipid peroxidation can compromise intestinal barrier integrity and promote the translocation of microbial products such as LPS, whereas dysbiosis-associated depletion of SCFAs and tryptophan-derived metabolites weakens antioxidant defenses and sustains activation of the Th17/IL-23 axis. Together, these reciprocal interactions integrate redox imbalance, immune dysregulation, and microbial alterations into a self-amplifying pathogenic loop that underlies psoriatic inflammation.

### 3.2. Pemphigus Vulgaris

Pemphigus vulgaris (PV) is a severe autoimmune blistering disorder characterized by the loss of keratinocyte adhesion, known as acantholysis, leading to intraepidermal blister formation affecting both the skin and mucous membranes [54]. The disease is primarily mediated by pathogenic IgG autoantibodies targeting desmosomal cadherins, particularly desmoglein 3 (Dsg3) and, to a lesser extent, desmoglein 1 (Dsg1), which are essential for maintaining intercellular cohesion within the epidermis [55].

**Figure 2:**
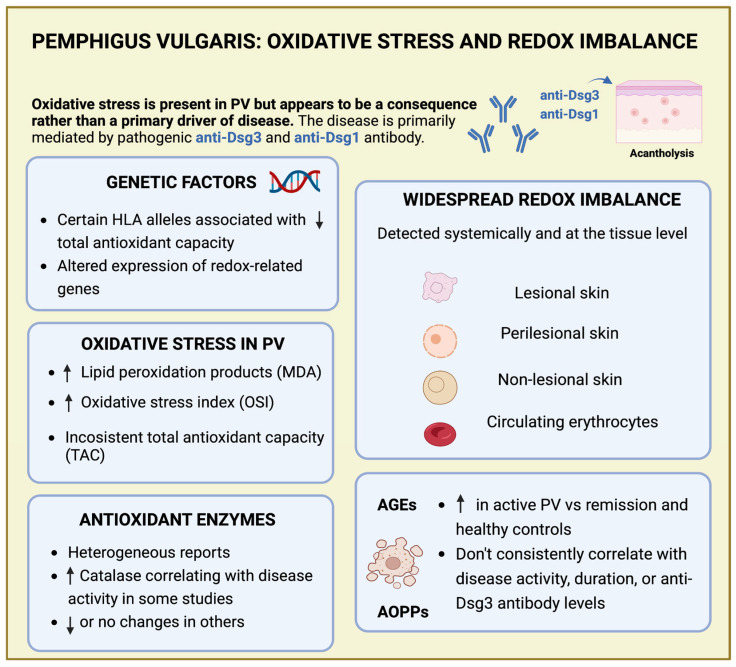
An overview of the main mechanisms related to oxidative stress involved in the pathogenesis of Pemphigus Vulgaris. Symbols: ↑, increased; ↓ reduced. Created in BioRender. Gama de Souza Silva, M. C. (2026) https://BioRender.com/rveb0dv (accessed on 13 May 2026).

Although the pathogenic role of anti-desmoglein antibodies is well established, antibody titers do not consistently correlate with disease severity, and patients in clinical remission may still exhibit circulating autoantibodies [56,57]. These observations indicate that PV pathogenesis cannot be fully explained by autoantibody production alone, and that additional mechanisms, including genetic predisposition, environmental triggers, and OS, may contribute to disease onset and progression (Figure 2).

In this context, PV-associated IgG has been proposed to induce OS within keratinocytes. Experimental studies have demonstrated that binding of autoantibodies to Dsg3 can increase intracellular ROS, thereby contributing to the disruption of intercellular junctions and promoting acantholysis [58,59]. OS may directly impair cell–cell adhesion via cadherin internalization and cytoskeletal reorganization [59]. Thus, the oxidative response induced in keratinocytes represents a relevant link between autoantibody binding, desmosomal instability, and epidermal blistering.

At the clinical level, studies support the presence of OS in PV (Table S1). Elevated lipid peroxidation products such as MDA have been reported, indicating increased oxidative damage to cellular membranes [60]. Similarly, increased oxidative stress index (OSI) values have been observed, although findings regarding total antioxidant capacity (TAC) remain inconsistent [61]. Antioxidant enzyme activity has also shown heterogeneous results: some studies reported increased catalase activity in correlation with disease activity, whereas others found reduced or unchanged enzymatic activity [60,62].

More stable biomarkers, including AGEs and AOPPs, indicate cumulative oxidative damage and have been found elevated in patients with active PV compared to those in remission and healthy controls [63,64]. Interestingly, these markers do not consistently correlate with disease activity, duration, or anti-Dsg3 antibody levels, supporting the possibility that OS may reflect a secondary phenomenon rather than a primary driver of disease.

Genetic factors also modulate OS in PV. Studies suggest that certain HLA alleles are associated with reduced total antioxidant capacity and altered expression of redox-related genes, highlighting the influence of genetic predisposition on redox balance [50]. Importantly, this imbalance has been detected both systemically and at the tissue level, including lesional, perilesional, and non-lesional skin, as well as circulating erythrocytes, indicating that oxidative alterations in PV are not confined to clinically active lesions [61].

Emerging evidence links alterations in the gut microbiota (GM) to PV pathogenesis (Table S2). High-throughput sequencing studies have revealed a possible compositional shift at the genus and species levels, including depletion of SCFA-producing bacteria such as *Lachnospiraceae incertae sedis*, *Coprococcus*, *Faecalibacterium*, and *Roseburia* [55,56]. These taxa maintain intestinal barrier integrity and modulate immune homeostasis. Conversely, potentially pathogenic taxa like *Escherichia coli*, *Klebsiella*, *Enterobacter*, *Granulicatella*, and *Flavonifractor* are often enriched in PV patients [55,57]. Therefore, PV-associated dysbiosis appears to involve both the reduction in taxa with immunoregulatory functions and the enrichment of bacteria with potential pro-inflammatory relevance.

Gut dysbiosis may promote increased intestinal permeability (“leaky gut”) and enhanced translocation of microbial products, such as LPS, triggering systemic immune activation and sustaining autoimmunity [58]. In addition to compositional changes, metabolomic studies have revealed alterations in lipid metabolism, including elevated phosphatidylethanolamine levels, which may contribute to inflammasome activation and inflammatory responses [56,58].

While alpha diversity findings are inconsistent, PV patients appear to exhibit reduced microbial richness, increased *Proteobacteria*, and decreased *Firmicutes*, particularly SCFA-producing families [55,60]. Certain taxa have been reported to correlate with autoantibody profiles: for instance, reduced *Veillonella* associates with higher anti-Dsg3 antibodies, while *Prevotella* and *Coriobacteriaceae* family members relate to anti-Dsg1 antibodies [55,56]. Furthermore, beneficial SCFA-producing genera negatively correlate with inflammatory cytokines (IL-6, IL-8, IL-17A), supporting their protective role in immune regulation [60].

Therapeutic interventions, including systemic glucocorticoids, can partially modify microbial composition, reducing pathogenic taxa such as Escherichia coli while leaving overall diversity largely unchanged [58]. These treatment-associated changes further indicate that immune modulation and microbiome composition are interconnected in PV, although the clinical relevance of these changes remains to be clarified.

The interplay between OS and gut microbiota in PV likely contributes to disease progression. OS may exacerbate immune dysregulation, while microbial dysbiosis can impair barrier function, promote systemic inflammation, and influence autoantibody production. Together, these factors create a self-perpetuating feedback loop sustaining tissue damage and disease activity.

### 3.3. Atopic Dermatitis

Atopic dermatitis (AD), also referred to as atopic eczema (AE), is a chronic inflammatory skin disease characterized by xerosis, intense pruritus, eczematous lesions, and a substantial impact on quality of life. It commonly begins in early childhood and often represents the first manifestation of the atopic march, preceding or accompanying food allergy, asthma, and allergic rhinitis [65].

AD pathogenesis is multifactorial and results from the interaction between genetic susceptibility, oxidative stress, environmental triggers, epidermal barrier dysfunction, type 2 immune polarization, and microbial imbalance. Epidermal barrier impairment represents a central event in AD. Loss-of-function mutations or reduced expression of filaggrin (FLG) compromise stratum corneum integrity, reduce natural moisturizing factor generation, and facilitate allergen penetration and microbial colonization [66]. In this context, *S. aureus* colonization plays a pivotal role by aggravating barrier disruption, promoting inflammation, and inducing itch–scratch-mediated damage through mechanisms involving the V8 protease–PAR1 axis [67]. Thus, epidermal barrier impairment, microbial colonization, pruritus, and mechanical injury are closely interconnected and contribute to the persistence of AD lesions.

**Figure 3:**
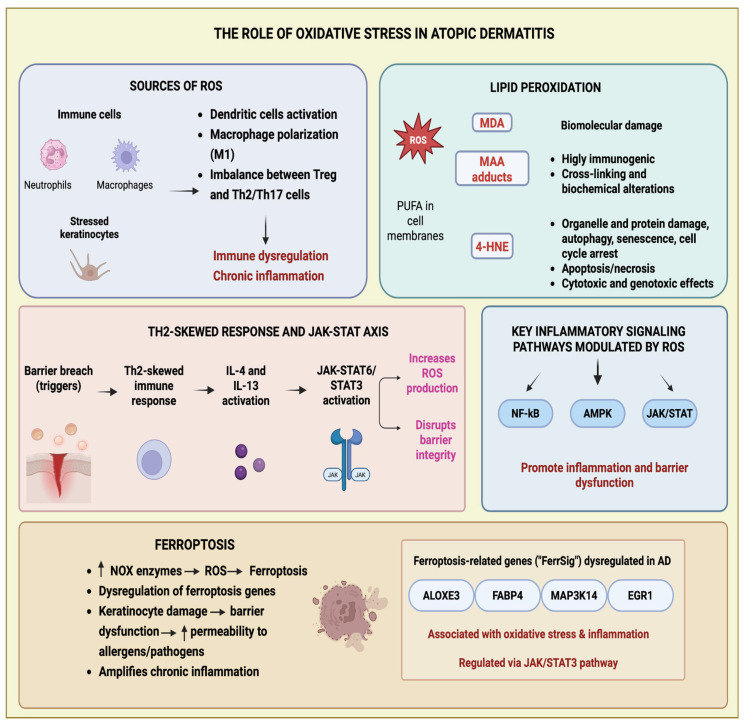
An overview of the main mechanisms related to oxidative stress involved in the pathogenesis of Atopic Dermatitis. Symbols: ↑, increased; ↓ reduced. Created in BioRender. Gama de Souza Silva, M. C. (2026) https://BioRender.com/1yxf0iq (accessed on 13 May 2026).

In addition to barrier defects, OS appears to play a crucial role in AD pathogenesis (Table S1). ROS are generated by stressed keratinocytes and activated immune cells, including neutrophils and macrophages. Under physiological conditions, ROS contribute to host defense and cellular signaling; however, excessive ROS production damages proteins, lipids, and DNA, promotes lipid peroxidation, and weakens epidermal barrier integrity [68] (Figure 3). This redox imbalance reinforces a pathogenic loop in which epithelial damage promotes inflammation, while inflammatory cytokines further enhance oxidative stress.

Keratinocytes play a central role in epidermal barrier homeostasis by regulating structural proteins such as filaggrin (FLG), loricrin (LOR), and involucrin (IVL). FLG degradation products, including urocanic acid, contribute to stratum corneum hydration and pH regulation. In AD, excessive ROS production disrupts these processes, weakening barrier integrity and amplifying inflammatory signaling.

AHR activation normally supports keratinocyte differentiation and promotes the expression of barrier-related proteins, including FLG, LOR, and IVL [69]. However, this protective axis is counterbalanced by type 2 cytokine signaling. IL-4 and IL-13 activate the JAK–STAT6/STAT3 pathway, suppressing epidermal differentiation, impairing barrier integrity, and promoting oxidative stress [70]. In turn, oxidative stress modulates inflammatory pathways such as NF-κB, AMPK, and JAK/STAT, reinforcing cytokine production and epithelial inflammation [71].

This creates a self-amplifying loop in which barrier disruption promotes Th2-skewed inflammation, while IL-4/IL-13 signaling enhances ROS generation and further compromises keratinocyte function. Consistently, STAT3 inhibition reduces cytokine-induced intracellular ROS in keratinocytes [72]. MicroRNA-mediated regulation, including the miR-1294/STAT3/NF-κB axis, may further modulate inflammation, oxidative stress, and barrier repair [71].

Beyond keratinocytes, oxidative stress promotes dendritic cell activation, M1 macrophage polarization, and an imbalance between regulatory and effector T cells, thereby sustaining immune dysregulation and chronic inflammation in AD [73].

Lipid peroxidation represents a major consequence of OS. Reactive species target PUFAs in cell membranes, generating lipid hydroperoxides and byproducts such as MDA, and 4-hydroxynonenal (4-HNE), two major secondary products of oxidative lipid damage [74].

Once produced, MDA can react with cellular proteins and DNA to form adducts, including advanced lipid peroxidation end products (ALPEs), thereby contributing to biomolecular damage and inflammatory amplification [75,76]. Under oxidative conditions, MDA metabolism may also generate malondialdehyde-acetaldehyde (MAA) adducts, which are highly immunogenic and can promote cross-linking reactions with relevant biochemical consequences [77,78,79].

Similarly, 4-HNE exerts concentration-dependent effects: at low levels, it may participate in adaptive antioxidant responses, whereas higher levels promote organelle and protein damage, senescence, cell-cycle arrest, apoptosis, or necrosis [74,80]. These mechanisms suggest that excessive lipid peroxidation directly links ROS accumulation to keratinocyte dysfunction, barrier impairment, and chronic inflammation in AD.

A case–control study by Amin et al., including 65 patients with AD and 65 healthy controls, further supported the presence of systemic oxidative imbalance in AD. The authors reported significantly higher serum levels of MDA, indicating increased lipid peroxidation, together with reduced antioxidant concentrations in patients compared with controls. Significant differences in serum macro- and trace-element profiles were also observed, including sodium, potassium, calcium, zinc, and iron. Overall, these findings suggest that AD is associated with enhanced oxidative damage and impaired antioxidant defense mechanisms, potentially contributing to disease-related inflammation and barrier dysfunction [81].

As for psoriasis, recent evidence also shows that ferroptosis may contribute to keratinocyte damage and barrier dysfunction. Dysregulation of ferroptosis-related genes, including GPX4 and SLC7A11, has been observed in AD and may promote lipid peroxidation, reduce keratinocyte viability, increase epidermal permeability, and facilitate allergen and pathogen penetration [82]. Furthermore, NADPH oxidase (NOX) enzymes, which produce ROS, are up-regulated in AD keratinocytes, thereby promoting ferroptosis and possibly further compromising the epidermal barrier [83].

Ferroptosis-related genes, including ALOXE3, FABP4, MAP3K14, and EGR1, have emerged as significantly dysregulated molecular signatures in AD skin lesions. This gene set, referred to as “FerrSig,” appears to link ferroptosis with OS and inflammation in AD, potentially through regulation by the JAK/STAT3 pathway [84]. Overall, these findings suggest that ferroptosis may represent a redox-dependent mechanism through which oxidative injury contributes to keratinocyte dysfunction, barrier impairment, and inflammatory persistence in AD. In parallel, alterations in the skin microbiome further contribute to disease pathogenesis and prognosis (Table S2). Evidence indicates that bacterial diversity decreases at predilection sites, with an increased abundance of *Staphylococcus* species, particularly *S. aureus*. The colonization rate of *S. aureus* was found to be approximately 70% in lesional skin and 39% in non-lesional skin [85]. In contrast, coagulase-negative *S. epidermidis*, a commensal bacterium that forms biofilms through the production of polysaccharide intercellular adhesins (PIA), inhibits pathogenic colonization and reduces inflammation by regulating the TLR3 signaling pathway [86]. Furthermore, *S. epidermidis* likely increases FLG expression and produces the natural moisturizing factor (NMF), thereby improving the compactness of the stratum corneum and, consequently, the skin barrier function [84]. Mutations in FLG cause barrier defects that facilitate the colonization of *S. aureus* and promote Th2-type inflammation [87]. 

*Staphylococcus hominis* (*S. hominis*), another plasma coagulase-negative bacterium, secretes autoinducer peptides to inhibit the expression of the harmful protease EcpAd and prevent the proliferation of pathogenic bacteria [88]. Local microenvironmental factors, including humidity, pH, temperature, and lipid content, further shape microbial composition. For instance, high humidity may favor *S. epidermidis* growth and ceramide production, while increased skin pH facilitates *S. aureus* survival and expansion [20,89].

Sebum-derived free fatty acids exert antimicrobial activity and provide substrates for *Cutibacterium acnes*, which contributes to SCFA production and pH regulation.

In AD lesions, this balanced microbial ecosystem is disrupted. *S. aureus* becomes predominant and releases virulence factors, including δ-toxin, superantigens, and other toxins, which impair keratinocyte tight junctions and amplify inflammation. At the same time, the reduction in commensal species such as *S. epidermidis* and *C. acnes* weakens microbial competition and reduces the production of protective metabolites, including anti-inflammatory SCFAs [90]. In skin folds, AD-associated dysbiosis is also characterized by an altered *Corynebacterium*/*Staphylococcus* ratio, a shift that has been linked to impaired antimicrobial peptide production driven by Th2 cytokines, particularly IL-4 and IL-13 [91]. These microbiome alterations further compromise the epidermal barrier by increasing skin pH and transepidermal water loss (TEWL). In response to barrier disruption and microbial stimuli, keratinocytes release epithelial alarmins such as TSLP, IL-1β, and IL-8, which recruit inflammatory cells, including IL-17+ neutrophils, and reinforce type 2 immune responses involving IL-4, IL-5, IL-13, and IL-22. Moreover, *S. aureus*-derived superantigens and toxins activate basophils and mast cells, promoting histamine release and exacerbating pruritus and inflammation [92]. Thus, cutaneous dysbiosis in AD contributes to a self-sustaining loop linking microbial imbalance, barrier dysfunction, itch, and immune activation.

Gut-skin axis (GSA) imbalance can also influence skin inflammation through immune pathways, thus exacerbating AD symptoms.

Reduced microbial diversity and richness in early life are associated with impaired immune system maturation, characterized by insufficient induction of Th1 cells and an inability to suppress Th2 responses. Furthermore, evidence suggests that there is a correlation between reduced alpha diversity in the gut microbiota and greater AD severity [93,94,95].

These observations align with the hygiene hypothesis, according to which insufficient microbial exposure during early life may compromise immune education and favor allergic disease development. Consistently, infants with AD frequently exhibit gut microbiota alterations characterized by reduced abundance of immunoregulatory and SCFA-producing taxa, including *Lactobacillus* spp., *Bifidobacterium* spp., *Akkermansia muciniphila*, *Faecalibacterium prausnitzii*, and *Coprococcus eutactus* [93]. Several studies have reported a reduced abundance of *Lactobacillus* species in pediatric patients with AD compared to healthy controls, likely due to a weakened intestinal epithelial barrier, which seems to promote systemic inflammation through the translocation of antigens and toxins from the intestinal lumen [96]. It has been reported that patients with AD have lower concentrations of *Bifidobacterium* spp., and that levels of *B. breve* and *B. longum* are positively correlated with symptom severity [97,98]. In fact, *B. longum* has been shown to likely alleviate AD symptoms by increasing the metabolism of tryptophan and indole-3-carbaldehyde (I3C), thereby activating the AHR pathway and suppressing Th2 immune responses [99]. Conversely, a higher abundance of potentially pro-inflammatory taxa, such as *Escherichia coli* and *Clostridioides difficile*, has been linked to AD onset and severity. Since *E. coli* is among the earliest colonizers of the neonatal gut, its early expansion may influence immune maturation. Consistently, a large birth cohort study reported increased *E. coli* abundance at one month of age in infants who later developed AD [100,101]. *C. difficile* has also been found more frequently in children with AD and may sustain chronic inflammation through toxin-mediated epithelial damage and impairment of intestinal barrier integrity [97].

The gut microbiota contributes to regulating the immune system by producing SCFAs such as butyric acid, propionic acid, and acetic acid [98]. These metabolites support epithelial barrier integrity and exert anti-inflammatory effects by promoting regulatory T-cell activity and suppressing cytokines such as IL-6 and TNF-α [102]. In AD, reduced abundance of SCFA-producing bacteria, including *Bifidobacterium*, *Blautia*, *Coprococcus*, *Eubacterium*, and *Propionibacterium*, may impair this protective metabolic axis, favoring intestinal barrier dysfunction, increased permeability, and systemic inflammation [103]. Several studies have linked altered SCFA profiles to AD severity and early disease development. Reduced bacterial butyrate production has been associated with disease severity [95], and longitudinal infant studies reported lower levels of butyrate, valerate, and propionate in children with AD during early life [104,105].

Nevertheless, SCFA levels are influenced by diet, age, disease activity, and methodological variability, highlighting the need for standardized sampling, analytical procedures, and data processing across future studies [106].

Various therapeutic strategies targeting the microbiome have shown promising results. Prebiotics and probiotics may reduce intestinal and systemic inflammation, enhance regulatory immune responses, and improve epithelial barrier function, although their clinical efficacy appears to be strain-specific and heterogeneous [107]. Biologic drugs such as dupilumab have been shown to improve microbiota dysbiosis, with increased levels of *Bifidobacterium* and SCFA-producing bacteria, regardless of their effects on skin inflammation [108]. These findings suggest that immune modulation and microbial composition may be reciprocally connected.

Additionally, fecal microbiota transplantation (FMT) is a therapeutic option to restore diversity in the gut microbiota. It involves transplanting fecal microbiota from a healthy donor into the gut of a patient suffering from dysbiosis to restore the diversity and function of the gut microbiota, thereby influencing AD via the GSA [109]. In a randomized, double-blind study involving adults with moderate-to-severe AD, FMT was associated with significant improvement in Eczema Area and Severity Index (EASI) scores, with a higher proportion of patients achieving EASI-50 compared with placebo. These clinical benefits were accompanied by increased abundance of *Megamonas fusiformis*, reduced Th2 and Th17 cell responses, and lower serum levels of TNF-α and total IgE [110]. In parallel, preclinical data from an AD mouse model showed that FMT increased SCFA production, particularly butyrate, thereby improving skin barrier function and attenuating inflammatory responses [111].

Washed microbiota transplantation (WMT), an improved FMT method that reduces infection risk by purifying donor feces, showed a significant reduction in SCORAD scores and improvement in gut and skin microbiota in adolescent AD patients, with no adverse events [112]. However, despite these promising findings, current evidence remains preliminary and requires validation in larger, well-controlled studies with standardized protocols and longer follow-up [113].

Overall, AD exemplifies the close interconnection between oxidative stress, barrier dysfunction, immune polarization, and microbiome dysbiosis. ROS overproduction and lipid peroxidation impair keratinocyte function and amplify Th2-skewed inflammation, while skin and gut dysbiosis reduce commensal protection, weaken epithelial integrity, and impair SCFA-mediated immune regulation. These reciprocal interactions generate a self-sustaining inflammatory loop in which redox imbalance and microbial alterations reinforce each other, contributing to disease initiation, persistence, and severity. Future therapeutic strategies may benefit from integrating clinical phenotype, redox biomarkers, microbiome profiling, and metabolic signatures to identify patients who may benefit from personalized antioxidant or microbiome-targeted interventions.

### 3.4. Urticaria

Urticaria is a skin disorder characterized by erythematous, edematous, and pruritic lesions [114]. Chronic urticaria is defined as the continuous or intermittent presence of urticaria for more than 6 weeks and may sometimes be accompanied by angioedema. Among patients with chronic urticaria, a substantial proportion (66–93%) have chronic spontaneous urticaria (CSU), for which the precise pathogenesis remains largely unknown. CSU is characterized by immune-mediated inflammatory mechanisms [115].

The role of OS in CSU remains debated, although alterations in systemic and cellular redox markers have been reported [116].

**Figure 4:**
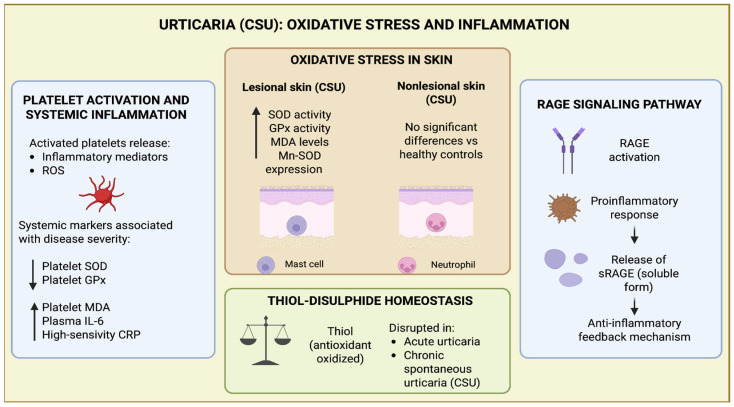
An overview of the main mechanisms related to oxidative stress involved in the pathogenesis of Urticaria (CSU). Symbols: ↑, increased; ↓ reduced. Created in BioRender. Gama de Souza Silva, M. C. (2026) https://BioRender.com/zjyfpel (accessed on 13 May 2026).

In CSU, oxidative and inflammatory pathways are likely to be interconnected (Table S1) (Figure 4). Given that platelets release inflammatory mediators and ROS upon activation, studies have assessed platelet oxidative status, including MDA, SOD, and GPx, together with systemic inflammatory markers such as plasma IL-6 and high-sensitivity C-reactive protein, and their association with disease severity [115]. At the skin level, OS involvement in CSU has been evaluated by measuring key oxidative markers, including SOD and GPx activities and MDA levels in lesional and nonlesional skin. Results showed that these parameters were markedly increased in lesional skin compared with healthy subjects, whereas no differences were observed between non-lesional skin from CSU patients and controls; manganese-dependent SOD expression was upregulated in lesional skin. These findings suggest that OS is increased in lesional tissue and may represent a consequence of local inflammation [117]. RAGE signaling has also been implicated in inflammation-related pathways.

Growing evidence suggests that the gut microbiome plays a role in the pathogenesis of allergy and autoimmunity (Table S2); however, the association between abnormalities in the gut microbiota and CSU remains largely undefined [118]. Subsequent work has examined alterations in the gut microbiota in clinical subsets, such as H1-antihistamine-resistant CSU, and has linked these alterations to systemic inflammation [119]. Integrated analyses combining 16S rRNA gene sequencing and untargeted serum metabolomics have further explored the relationship between gut microbes and circulating metabolites in CSU. These studies reported decreased alpha diversity in CSU, increased abundance of unidentified *Enterobacteriaceae*, and reduced abundance of *Bacteroides*, *Faecalibacterium*, *Bifidobacterium*, and unidentified *Ruminococcaceae*. Serum metabolomics showed altered levels of docosahexaenoic acid, arachidonic acid, glutamate, and succinic acid, suggesting changes in unsaturated fatty acid and butanoate metabolic pathways; docosahexaenoic acid and arachidonic acid were positively correlated with *Bacteroides*. Overall, these findings indicate that microbial and metabolic alterations coexist with inflammatory and immune abnormalities in CSU, although their functional relevance remains to be fully clarified [120]. Mendelian randomization (MR) studies have addressed potential causal links between microbiota taxa and urticaria/CSU. An MR study in East Asians was designed to clarify causal and mediating relationships among gut microbiota, blood metabolites, and urticaria [121].

Beyond CSU, a study comparing the intestinal microbiota of patients with urticaria and healthy controls investigated the role of *Blastocystis*. Significant differences were observed in selected taxa (including *Proteobacteria*, *Bacteroidetes*, *Escherichia*, *Phocaeicola*, and *Prevotella*) between urticaria and control groups; *Bacteroidetes* and *Phocaeicola* also differed between samples with and without *Blastocystis*, and overall microbiota composition differed between *Blastocystis*-positive and -negative samples. The *Firmicutes*/*Bacteroidetes* ratio was reported as 4.1 in controls and 6.4 in patients with urticaria. These results indicate that both urticaria and *Blastocystis* colonization are associated with changes in intestinal microbiota composition [114].

Taken together, current evidence indicates that CSU is associated with both redox alterations and changes in gut microbiota composition and metabolism. Increased oxidative markers in lesional skin, platelet-related oxidative parameters, reduced alpha diversity, depletion of selected SCFA-producing taxa, and changes in circulating metabolites such as arachidonic and docosahexaenoic acids support the involvement of interconnected inflammatory, oxidative, and microbial pathways. However, available data remain largely associative, and further studies are needed to clarify whether these alterations contribute directly to disease persistence or mainly reflect ongoing inflammation.

### 3.5. Alopecia

Alopecia areata (AA) is an autoimmune disorder of the hair follicle, clinically characterized by non-scarring hair loss with preservation of follicular structures. Hair loss may range from well-defined patches to diffuse or total alopecia and can affect all hair-bearing sites, although patchy scalp involvement is the most common presentation. Breakdown of hair follicle immune privilege is considered a pivotal event in disease pathogenesis [122].

Genetic and experimental data indicate that AA is a complex, polygenic disorder. Several susceptibility loci have been identified in pathways involved in hair follicle cycling and development, supporting a model in which genetic predisposition interacts with immune dysregulation and environmental triggers to drive a chronic inflammatory process [123]. Although diagnosis is usually based on characteristic clinical manifestations, dermoscopy and histopathology may serve as useful adjuncts in atypical or unclear cases [122].

**Figure 5:**
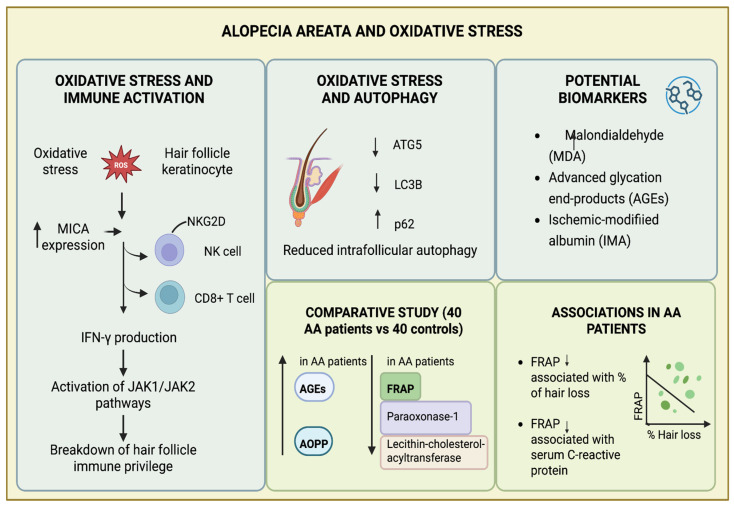
An overview of the main mechanisms related to oxidative stress involved in the pathogenesis of Alopecia areata. Symbols: ↑, increased; ↓ reduced.Created in BioRender. Gama de Souza Silva, M. C. (2026) https://BioRender.com/2y4kxi9 (accessed on 13 May 2026).

Within this pathogenetic framework, evidence indicates that OS contributes to AA pathophysiology (Table S1) (Figure 5). Altered OS markers have been detected in both serum and skin samples from patients with AA, supporting the presence of a pro-oxidative state. OS has been shown to induce MHC class I chain-related A (MICA) expression in hair follicle keratinocytes, with subsequent activation of the NKG2D receptor on NK cells and cytotoxic CD8+ T cells. This interaction promotes IFN-γ production and activates JAK1 and JAK2 signaling, thereby destabilizing the immune-privileged status of the hair follicle. OS also activates the KEAP1–NRF2 pathway, and reduced intrafollicular autophagy during AA progression has been associated with decreased ATG5 and LC3B and increased p62 in the hair matrix [124].

Together, these mechanisms connect oxidative imbalance with immune activation, altered follicular homeostasis, and hair follicle damage.

Multiple OS-related biomarkers have been investigated in AA, including MDA, AGEs, ischemia-modified albumin (IMA), and AOPPs, as well as antioxidant-related measures such as paraoxonase-1, lecithin-cholesterol acyltransferase, and ferric-reducing antioxidant power (FRAP) [124,125]. In a comparative cross-sectional study of patients with AA and healthy controls, AGEs and AOPP were significantly higher in AA, whereas FRAP, paraoxonase-1, and lecithin-cholesterol acyltransferase were significantly lower. FRAP levels correlated with the percentage of hair loss and with serum C-reactive protein, supporting impairment of the oxidant–antioxidant enzymatic system in AA. However, the cross-sectional design and relatively small sample size limit causal inference [125].

The link between OS, MICA–NKG2D activation, and JAK–STAT signaling provides a rationale for targeting these pathways. JAK inhibitors have emerged as a major therapeutic advance in AA, and there is growing interest in nutraceuticals that modulate OS balance as potential adjuncts to standard treatments [124].

Beyond OS, emerging data suggest the role of the microbiome in AA, although current evidence supports association rather than causation. AA is characterized by the collapse of hair follicle immune privilege and local immune cell infiltration, but the upstream drivers of this immune dysregulation remain incompletely defined. Several studies suggest that both cutaneous and gut dysbiosis may contribute to immune imbalance, particularly through effects on barrier function and regulatory Treg [126,127,128].

The gut microbiota has been proposed as a potential modulator of AA via the GSA (Table S2). Gut dysbiosis may disrupt intestinal barrier integrity and immune tolerance by altering Treg populations and promoting systemic inflammation, thereby potentially contributing to disease onset and progression. AA has also been linked to OS, autoimmune phenomena, neuropsychological factors, pathogens, immune checkpoint inhibitors, and microecological imbalance in genetically susceptible individuals, suggesting a complex network of interacting triggers [127,128].

At the cutaneous level, scalp microbiome studies indicate compositional shifts in AA [127]. One study reported a genus-level increase in *Propionibacterium* with a concomitant decrease in *Staphylococcus* in the AA-affected scalp. At the species level, *Propionibacterium acnes* was significantly increased, and *S. epidermidis* was significantly decreased, whereas *S. aureus* showed no significant difference. Sequencing profiles also demonstrated distinct microbial communities across scalp compartments, from the epidermis to the hypodermis, with clear differences between healthy and AA-affected scalp [129]. Additional work linking scalp microbiota imbalance with AA severity and systemic inflammatory markers supports an association between local dysbiosis and both local and systemic inflammation, although causality remains unproven [129].

Studies of the gut microbiome in AA report more subtle but potentially relevant changes. In an analysis of gut microbiota composition, combined with fecal and urinary volatile organic compound metabolites, overall microbiota composition did not differ markedly between AA individuals and controls; however, AA subjects exhibited reduced species richness and evenness. Differential abundance analysis identified taxa enriched in AA, including Firmicutes, Lachnospirales, and *Blautia*, and in healthy individuals, including *Coprococcus*. Integration of microbiome and metabolome data with DIABLO confirmed these compositional shifts and highlighted metabolite biomarkers, including esters of branched-chain fatty acids and branched-chain amino acids, as potential predictors of AA [130]. Narrative and systematic reviews emphasize that 16S rRNA next-generation sequencing and metagenomic approaches have enabled progressively finer characterization of both gut and skin microbiota in AA and support the concept that microecological imbalance may modulate disease risk and course [126,131].

Overall, available data suggest that AA is a multifactorial autoimmune disease arising from the interplay of genetic susceptibility, breakdown of hair follicle immune privilege, immune dysregulation, OS, and cutaneous/gut dysbiosis.

In conclusion, current evidence supports a reciprocal relationship between OS and GSA dysbiosis in AA. ROS-driven disruption of hair follicle immune privilege and activation of the MICA–NKG2D and JAK–STAT pathways may be amplified by dysbiosis-associated impairment of regulatory T-cell function and altered microbial metabolite profiles. Conversely, systemic immune activation and gut-derived inflammatory signals may exacerbate OS within the follicular microenvironment, thereby sustaining cytotoxic immune responses against the hair follicle. This reciprocal interplay integrates redox imbalance, immune dysregulation, and microecological alterations into a self-perpetuating pathogenic loop that may contribute to disease persistence and progression.

### 3.6. Vitiligo

Vitiligo is a chronic depigmenting disorder characterized by well-demarcated white patches resulting from selective melanocyte loss. Global prevalence is estimated at approximately 0.5–2% [132]. Vitiligo is currently framed as an immune-mediated/autoimmune disease with multifactorial pathobiology, in which immune mechanisms interact with inflammatory, metabolic, and oxidative-stress-related processes [133,134].

From an immunological perspective, a central feature is the activation of autoreactive CD8+ T cells in the skin. This activation is associated with IFN-γ release, which can upregulate keratinocyte chemokines such as CXCL9 and CXCL10, promoting further recruitment of effector T cells and sustaining melanocyte-directed immune attack [134]. These immune circuits may also be influenced by systemic modulators, including OS and the microbiome, which are increasingly considered relevant to disease heterogeneity and potential adjunctive therapeutic approaches.

**Figure 6:**
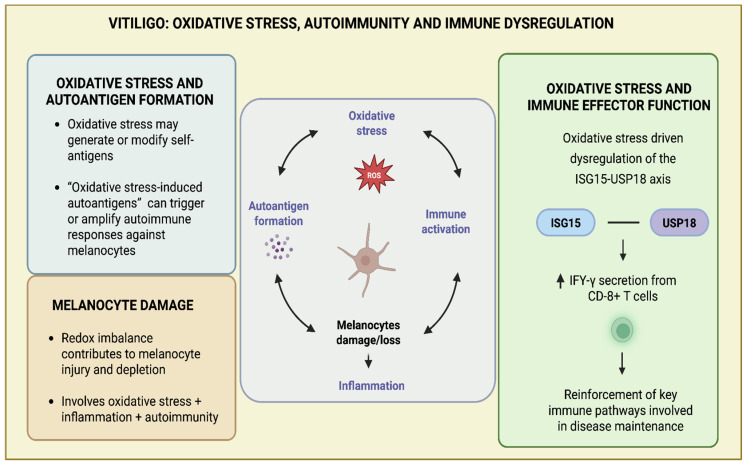
An overview of the main mechanisms related to oxidative stress involved in the pathogenesis of Vitiligo. Symbols: ↑, increased; ↓ reduced. Created in BioRender. Gama de Souza Silva, M. C. (2026) https://BioRender.com/louzdi1 (accessed on 13 May 2026).

OS is consistently highlighted as a relevant process in vitiligo pathogenesis (Table S1) (Figure 6). One proposed mechanism is that redox imbalance contributes to the generation or modification of self-antigens, creating “OS-induced autoantigens” that may trigger or amplify autoimmune responses against melanocytes [135]. In this view, OS is not only a downstream consequence of tissue injury but may actively contribute to immune recognition and the perpetuation of melanocyte-directed autoimmunity.

Recent evidence describes vitiligo as a condition in which melanocyte depletion is mediated by the interplay of OS, inflammation, and autoimmunity [134]. A more direct functional link between OS and immune effector pathways has also been reported: oxidative-stress-driven dysregulation of the ISG15–USP18 axis promotes IFN-γ secretion from CD8+ T cells in vitiligo [136]. Given the centrality of IFN-γ-related circuits in vitiligo immunopathology, these findings support a model in which OS can reinforce key immune mediators that maintain the disease.

Overall, available data indicate that OS contributes to vitiligo through at least two complementary mechanisms: the generation of potentially immunogenic self-antigen modifications [135] and the enhancement of immune pathways such as IFN-γ production by CD8+ T cells [136]. These mechanisms are part of a broader multifactorial framework that also includes inflammation and autoimmunity [133].

The relationship between the gut microbiome and skin disease is often framed within the GSA, whereby intestinal microbial communities shape host metabolism, inflammation, and immune responses with downstream cutaneous effects [134]. Within this framework, vitiligo has been explored in both preclinical and human studies (Table S2).

In a mouse model, antibiotic-induced microbial imbalance was associated with the development of vitiligo, supporting a functional contribution of dysbiosis in experimental settings [137]. In humans, analysis of matched skin and gut microbiomes in patients with vitiligo revealed pronounced skin dysbiosis and its links to mitochondrial and immune alterations, indicating that microbial shifts coexist with other biologically relevant disease-associated changes [138].

A 16S rRNA sequencing study comparing stool samples from 49 patients with vitiligo and 49 healthy controls reported reduced gut microbial diversity and distinct overall community composition in patients, with additional age-stratified analyses in minors and adults, and a primary focus on stable non-segmental vitiligo.

At the phylum level, patients showed an altered microbial composition, including a reduced *Firmicutes*/*Bacteroidota* ratio compared with controls. At the genus level, *Bacteroides* and *Parabacteroides* were identified as disease-associated taxa and were positively correlated with the Vitiligo Area Scoring Index (VASI) and disease duration, suggesting links to clinical severity. These findings support an association between specific gut microbiota profiles and vitiligo, although causality remains unproven [134].

Available studies also outline immuno-metabolic mechanisms through which dysbiosis may influence immune regulation in vitiligo. Commensal-derived metabolites have been shown to promote pTregs generation [139], and SCFAs regulate colonic Treg homeostasis [140]. The microbiome and butyrate also modulate energy metabolism and autophagy in the mammalian colon [141], and SCFAs are implicated more broadly in inflammatory skin diseases [142].

These findings support biological plausibility for a gut microbiome–immune–skin link in vitiligo [134]. Overall, current evidence points to vitiligo as a multifactorial immune-mediated disease in which OS, immune dysregulation, and gut/skin dysbiosis may interact. However, most human data are associative, and longitudinal and interventional studies are required to clarify causal pathways and to define the therapeutic potential of microbiome-targeted and redox-modulating strategies.

Taken together, these findings support a bidirectional interaction between OS and dysbiosis of the GSA in vitiligo. ROS-driven redox imbalance and ISG15–USP18-mediated amplification of IFN-γ signaling in CD8+ T cells may promote melanocyte-directed autoimmunity. In parallel, alterations in the gut microbiota—characterized by reduced diversity and shifts in taxa such as *Firmicutes* and *Bacteroidota*—may impair SCFA-mediated regulatory T-cell homeostasis and favor a pro-inflammatory milieu. In turn, dysbiosis-linked immune activation and metabolic imbalance may further exacerbate cutaneous OS, reinforcing melanocyte loss. Together, these processes form a self-sustaining loop that may underlie disease persistence and heterogeneity in vitiligo.

## 4. Materials and Methods

**Figure 7:**
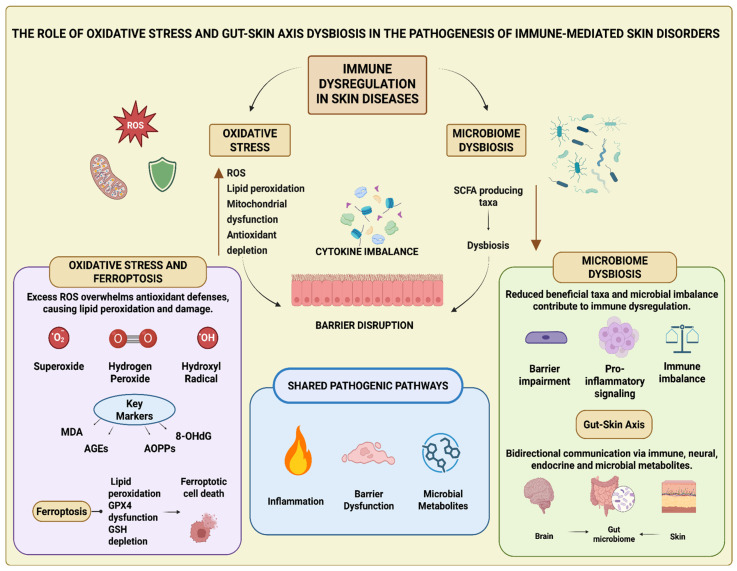
This schematic illustrates the bidirectional relationship between oxidative stress and microbiome dysbiosis as central drivers of immune dysregulation in inflammatory skin disorders. On the left, excessive production of ROS (superoxide anion, hydrogen peroxide, and hydroxyl radical) overwhelms antioxidant defenses, leading to lipid peroxidation, mitochondrial dysfunction, and depletion of endogenous antioxidants. These processes promote ferroptosis, a form of iron-dependent regulated cell death characterized by GSH depletion and impaired GPX4 activity. Key biomarkers of oxidative damage include MDA, AGEs, AOPPs, and 8-OHdG. On the right, gut and skin microbiome dysbiosis is characterized by a reduction in beneficial taxa, particularly SCFA–producing microorganisms, resulting in altered microbial composition and metabolic output. This imbalance contributes to impaired epithelial barrier integrity, increased pro-inflammatory signaling, and systemic immune imbalance. The gut–skin axis mediates bidirectional communication through immune, neural, endocrine, and microbial metabolite pathways linking the gut microbiota, central nervous system, and skin. Both oxidative stress and microbiome dysbiosis converge on shared pathogenic mechanisms, including cytokine imbalance, epithelial barrier disruption, and chronic inflammation. These interconnected pathways ultimately sustain immune dysregulation and contribute to the onset and progression of immune-mediated skin diseases. Symbols: ↑, increased; ↓ reduced.Created in BioRender. Gama de Souza Silva, M. C. (2026) https://BioRender.com/c18sjy5 (accessed on 13 May 2026).

We performed a comprehensive literature search in PubMed to identify relevant studies published over the last decade. The search strategy included the following keywords: “oxidative stress”, “lipid peroxidation”, “ferroptosis”, “reactive oxygen species”, “microbiome”, “skin diseases”, “inflammation”, “dysbiosis”. Only articles published in English were considered. Original research articles, reviews, and clinical studies investigating the roles of several OS mechanisms and microbiome alterations in immune-mediated skin disorders were considered, then sorted by relevance and screened for consistency with the aim of the review. Attention was given to studies exploring mechanistic pathways, clinical correlations, and potential therapeutic implications. The included studies were qualitatively analyzed and categorized by the specific oxidative pathways and microbiome alterations involved in the various dermatological conditions. The selected articles, along with their main findings and potential outcomes, are provided in Tables S1 and S2. A graphical representation of the highlighted interconnected redox and dysbiosis pathways across the major immune-mediated skin diseases is depicted in Figure 7.

## 5. Highlights

- OS, lipid peroxidation, ferroptosis, and elevated ROS drive tissue damage and sustain inflammation in autoimmune and inflammatory skin diseases.
- Microbiome dysbiosis interacts with OS to disrupt barrier function, amplify immune dysregulation, and modulate disease severity across skin disorders.

## 6. Conclusions

OS and microbiome dysregulation emerge as interconnected mechanisms across a spectrum of immune-mediated dermatoses. This review highlights that redox imbalance, manifested through ROS overproduction, lipid peroxidation, ferroptosis, and impaired antioxidant defenses, acts in concert with alterations in both the cutaneous and gut microbiomes to amplify immune dysregulation, compromise epithelial barrier integrity, and favor a chronic inflammatory state. The GSA represents a particularly relevant biological interface, whereby microbial dysbiosis disrupts intestinal permeability, depletes immunomodulatory metabolites, and shifts immune polarization toward pro-inflammatory Th1, Th2, and Th17 phenotypes, thereby affecting skin homeostasis. Conversely, OS can reshape microbial species and metabolite profiles, contributing to reciprocal interactions that may sustain tissue damage and disease activity. Despite the growing body of evidence, several limitations remain, mainly related to the cross-sectional design and small sample size of many available studies, as well as the heterogeneity of microbiome analyses. These factors make it difficult to clearly assess the relative contribution of OS and dysbiosis to disease initiation or perpetuation; therefore, causal inference remains limited. Future research should prioritize longitudinal and interventional studies, standardized microbiome profiling methods, and integrated multi-omics approaches. The highlighted shared pathogenic mechanisms identified across these clinically different conditions support important therapeutic implications. Strategies targeting redox homeostasis, including antioxidant supplementation, Nrf2 modulators, ferroptosis inhibitors, alongside microbiome-directed interventions such as probiotics, prebiotics, dietary modification, and fecal microbiota transplantation, represent a promising and yet underexplored frontier in dermatology. A deeper understanding of the OS-microbiome-axis may enable the development of precision therapeutic strategies tailored to the individual patient’s phenotype, microbiota composition, and redox pathways, moving dermatological care toward a more integrated and pathophysiology-guided management.

This review is limited by the heterogeneity of the included studies and the predominance of preclinical data. The lack of standardized methodologies, together with the cross-sectional design of most studies, further restricts the ability to draw causal inferences. In addition, many of the topics discussed are relatively recent and rapidly evolving areas of research, and some mechanisms therefore remain incompletely understood. These uncertainties reflect current gaps in the literature rather than limitations of the present analysis.

## Data Availability

No new data were created or analyzed in this study. Data sharing is not applicable to this article.

## Supplementary Materials

The following supporting information can be downloaded at: https://www.mdpi.com/article/10.3390/ijms27114656/s1.
