# Supplementary material for: Beyond Inflammation: The Role of Oxidative Stress and Gut–Skin Axis Dysbiosis in the Pathogenesis of Immune-Mediated Skin Disorders and Potential Therapeutic Implications
Source: Int J Mol Sci. 2026 May 22;27(11):4656. doi: 10.3390/ijms27114656 (PMC13256635; doi:10.3390/ijms27114656)
Supplement: Supplementary file 1 [file ijms-27-04656-s001.zip › ijms-4283336-supplementary.pdf]

Table S1. Summary of the main oxidative stress mechanisms involved in different skin disorders and their potential outcomes. Legend: PSO: psoriasis; PV: pemphigus vulgaris; AD: atopic dermatitis; U: urticaria; AA: alopecia areata; V: vitiligo.

Author (Year)	Disease	Type of Study/Study model	Oxidative stress pathway	Main Findings	Outcomes
Rendon & Schäkel (2019) [38]	PSO	Narrative review	Inflammation-driven ROS production via cytokine signaling (TNF-α, IL-17)	Immune-mediated disease driven by IL-23/Th17 axis	Framework for pathogenesis, not experimental
Medovic et al. (2022) [39]		Review	ROS activation of NF-κB, MAPK, and JAK/STAT pathways leading to chronic inflammation	Strong association between oxidative stress and inflammation in psoriasis	Suggests OS as therapeutic target
Lee et al. (2013) [40]		Animal study (knockout mice)	Loss of antioxidant defense (SOD deficiency) leads to ROS accumulation and IL-23/Th17 activation	Extracellular SOD deficiency increases IL-23-mediated inflammation	Supports causal role of ROS in inflammation
Oszukowska et al. (2020) [41]		Observational (clinical)	Impaired non-enzymatic antioxidant defense leads to increased oxidative damage	Reduced antioxidant levels (e.g., vitamin E) in patients	Suggests impaired antioxidant defense
Ten Bergen et al. (2020) [43]		Review	ROS-induced neoantigen formation leads to activation of adaptive immune response	Autoantigens (LL-37, ADAMTSL5) linked to immune activation	Links OS to autoimmunity
Hawkes et al. (2017) [44]		Review	Cytokine-driven inflammation amplifies ROS production (feed-forward loop)	IL-23/IL-17 axis central in disease	Mechanistic immune overview
Sikora et al. (2019) [45]		Observational (clinical biomarkers)	Inflammation secondary to gut barrier dysfunction induces ROS overproduction.	Increased intestinal permeability markers correlate with severity	Links systemic inflammation and barrier dysfunction
Nakai et al. (2009) [46]		Observational	ROS-induced oxidative DNA damage (↑8-OHdG).	Increased oxidative DNA damage marker (8-OHdG)	Confirms oxidative damage in patients
Shou et al. (2021) [49]		Experimental (molecular)	ROS-driven iron-dependent lipid peroxidation leading to ferroptosis (↓GPX4).	Ferroptosis markers upregulated in psoriasis	Suggests role of lipid peroxidation
Yesilova et al. (2013) [61]	PV	Case-control (clinical)	Global oxidative stress imbalance (↑OSI)	Increased oxidative stress index (OSI) in PV patients	Confirms systemic oxidative imbalance

Abida et al. (2012) [62]		Case-control (clinical)	Lipid peroxidation (↑MDA)	Elevated lipid peroxidation (MDA levels) in patients	Indicates membrane oxidative damage
Huang Y et al. (2021) [63]		Experimental (cellular/molecular)	Dysregulation of the YAP pathway induced by ROS contributes to acantholysis	PV IgG induces ROS and disrupts YAP signaling in keratinocytes	Antibody-mediated ROS generation contributes to acantholysis
Luo et al. (2025) [65]	AD	Review	ROS → DNA/protein/lipid oxidation	Antioxidants beneficial	Biomarkers + therapeutic targets
Hoyer et al. (2022) [66]		Prospective cohort + in vitro	Not primary (barrier defect)	FLG↓ → barrier↓ + S. aureus↑	Early AD risk
Deng et al. (2023**)** [67]		Experimental (in vivo + in vitro)	Not direct	V8 → PAR1 → itch	Anti-itch target
Furue et al. (2020) [70]		Review	IL-4/IL-13 → JAK/STAT → ROS	Th2 axis induces oxidative stress + barrier damage	Therapeutic targets (JAK, AHR)
Yan et al. (2022) **[71]**		In vitro + in vivo	Indirect (STAT3/NF-κB)	miR-1294↓ → STAT3↑ → inflammation	Improved barrier + reduced inflammation
Dao et al. (2020**)** [72]		In vitro (HaCat)	ROS scavenging + Nrf2 activation	↓NF-κB/STAT + ↑differentiation genes	Anti-inflammatory/antioxidant effect
Borgia et al. (2021) [73]		Review	Oxidative stress -> TSLP/IL-25/IL-33	Epithelial cytokines link OS–inflammation	Biomarkers/targets
Ayala et al. (2014) [74]		Review	Lipid peroxidation (MDA, 4-HNE)	Dual role (signaling/toxic)	Role in AD
Esterbauer et al. (1991) [75]		Review	Aldehydes from lipid peroxidation	Toxic second messengers	Biomolecular damage
Traverso et al. (2004) [76]		In vitro	MDA + ROS + metals	Protein adducts + peroxide formation	Tissue damage (aging)
Tuma et al. (2001) [77]		Biochemical (in vitro/in vivo)	MDA + acetaldehyde adducts	MDHDC formation via FAAB	Protein modification
Cheng et al. (2011**)** [79]		In vitro	ROS↑ + mitochondrial dysfunction	MDA → apoptosis/necrosis	Neuronal damage
Siems et al. (2003**)** [80]		Experimental	HNE detox (GSH pathways)	Rapid HNE degradation	Antioxidant defense
Amin et al. (2015**)** **[81]**		Case control	MDA↑ + antioxidants↓	Increased oxidative stress in patients	Association with eczema
Feng et al. (2022**)** **[82]**		In vivo + in vitro	Lipid peroxidation + iron	UV → ROS/lipid oxidation → ferroptosis sensitivity	Skin damage
Emmert et al. (2020**)** **[83]**		In vitro	NOX → ROS	NOX1/4 → ROS↑ + DNA damage	Antioxidant target
Rajappa M. et al. (2013) [115]	U	Cross-sectional	Nitric oxide (NO) / reactive nitrogen species (RNS)	↑ NO levels (or NOx metabolites) in CSU patients	Possible role of NO in disease activity and pathogenesis
Sagdic a. et al (2011) [116]		Case-control	Systemic oxidative stress (TOS/TAS, OSI)	↑ TOS and OSI, ↓ TAS in CSU patients	Confirms oxidative imbalance correlated with disease

Raho G. et al (2003) [117]		Experimental clinical-histological	Paraoxonase-1 (PON1) / lipid antioxidant system	↓ PON1 activity in CSU patients → reduced antioxidant defense	Involvement of oxidative stress in CSU pathogenesis
Peterle et al. (2023) [124]	AA	Review	OS → MICA upregulation → NKG2D activation → IFN-γ/JAK1/2	Pro-oxidative markers; reduced follicular autophagy	Links OS to immune activation and hair follicle damage
Shakoei S et al. (2023) [125]		Cross-sectional case–control study.	Increased oxidation products with reduced antioxidant capacity.		Confirms systemic oxidative imbalance in AA; severity association.
Xie H et al. (2016) [135]	V	Review	Oxidative stress → autoantigen formation	OS-induced changes generate or modify self-antigens, triggering autoimmune responses	Suggests OS contributes to initiation/amplification of melanocyte-directed autoimmunity
Lee EJ et al. (2024) [136]		Review	Experimental / Human	OS → ISG15–USP18 dysregulation → IFN-γ from CD8+ T cells	Links oxidative stress to immune activation and disease maintenance

Table S2. Summary of microbiota alterations and their implications in different skin disorders. Legend: PSO: psoriasis; PV: pemphigus vulgaris; AD: atopic dermatitis; U: urticaria; AA: alopecia areata; V: vitiligo.

**Author (Year)**	**Disease**	**Type of Study/Study model**	**Microbiota alteration/components**	**Main Findings**	**Outcomes**
Codoñer et al. (2018) [42]	PSO	Case-control (human microbiome)	Increased Firmicutes, decreased Bacteroidetes in gut; reduced diversity	Gut dysbiosis associated with disease severity	Supports dysbiosis in psoriasis
Zákostelská et al. (2016) [47]		Animal (FMT study)	Gut microbiota shifts in mice: ↑Lactobacillales, ↓Coriobacteriales & Clostridiales; affects skin inflammation.	Microbiota from diseased mice induces inflammation	Suggests causal role of microbiota
Assarsson et al. (2018) [52]		Interventional (phototherapy)	Lesional skin shows altered bacterial diversity and taxa shifts.	UVB alters skin microbiome composition	Therapy affects microbiome
Langan et al. (2019) [53]		Observational	Altered gut microbiome in psoriasis: ↑Firmicutes, ↓Bacteroidetes; associated with	Skin microbiome differs between lesional and non-lesional skin	Confirms cutaneous dysbiosis

			immune dysregulation.		
Scaglione et al. (2020) [55]	PV	Observational (human microbiome)	Altered gut, oral, and skin microbiota; compositional shifts	Microbiota differences across compartments	Supports multi-site dysbiosis
Wang et al. (2023) [56]		Observational + metabolomic	Altered gut microbiota with metabolic changes (lipid metabolites)	Microbiome and metabolites associated with disease onset and treatment response	Links microbiome to clinical course
Guo et al. (2023) [57]		Observational (multi-omics)	Dysbiosis with altered microbial composition and metabolites	Altered gut microbiome and metabolome in PV patients	Supports microbiome–metabolism interaction
Huang S et al. (2019) [58]		Observational (correlation study)	Increased Granulicatella, Flavonifractor; decreased SCFA-producers (Lachnospiraceae, Coprococcus)	Gut dysbiosis correlates with cytokine levels (IL-6, IL-17)	Links microbiota with immune activation
Li SZ et al. (2024) [59]		Observational	Increased Klebsiella, Akkermansia, Bifidobacterium, Prevotella; decreased Veillonella	Microbiota composition correlates with anti-desmoglein antibodies	Suggests microbiome–autoimmunity link
You S et al. (2024) [60]		Observational (clinical immunology)	Not microbiome focused, but a cytokine study	Altered cytokine/chemokine profiles in PV patients	Supports systemic immune dysregulation
Zheng et al. (2022) [20]	AD	Experimental (mouse model)	Skin microbiota – *S. epidermidis*	Produces sphingomyelinase → increases ceramides	Enhances skin barrier integrity and hydration
Kalankariyan et al. (2025) [84]		Computational/modeling study	Skin microbiota – *S. epidermidis*	Models interaction between commensals, immunity, and barrier function	*S. epidermidis* crucial for barrier homeostasis and immune modulation
Byrd et al. (2017) [85]		Longitudinal cohort + experimental (mouse model)	Skin microbiota – *S. aureus*, *S. epidermidis* (strain-level)	Severe AD linked to clonal *S. aureus* dominance; strain-specific inflammatory effects (TH2/TH17 activation)	Strain heterogeneity explains disease variability and severity
Lai et al. (2009) [86]		Experimental (in vitro + mechanistic)	Skin commensal staphylococci (LTA)	Lipoteichoic acid inhibits keratinocyte inflammation via TLR2 and TLR3 pathways	Microbiota can actively suppress skin inflammation
Paller et al. (2019) [87]		Narrative review	Skin microbiota (commensals vs pathogens)	Reduced diversity and increased *S. aureus* in AD; commensals may reduce severity	Microbiome-targeted therapies promising

Cau et al. (2021) [88]	Experimental (in vitro + mouse + human samples)	Skin microbiota – *S. epidermidis* (EcpA protease)	*S. epidermidis* can damage barrier via cysteine protease (EcpA)	Commensal may become pathogenic in AD
Scharschmidt et al. (2013) [89]	Review	Skin microbiota (general)	Dysbiosis contributes to inflammatory skin disease	Microbiome manipulation is therapeutic target
Almoughrabie et al. (2023) [90]	Experimental (in vitro + in vivo)	Skin microbiota– *Cutibacterium acnes*	SCFAs increase lipid synthesis via PPARα	Improved barrier and antimicrobial function
Tudela et al. (2021) [91]	Review	Gut microbiota (keystone species)	Keystone microbes maintain immune and metabolic balance	Targets for microbiome-based therapies
Alam et al. (2022) [92]	Review	Skin + gut microbiota	Dysbiosis contributes to AD; microbiota modulates immunity/barrier	Probiotics and microbiota-targeted therapies promising
Malgesini et al. (2026) [93]	Systematic review	Gut microbiota	Reduced diversity; ↓ *F. prausnitzii*, *Bifidobacterium*, *Akkermansia* in AD	Gut dysbiosis linked to inflammatory skin diseases
Wang et al. (2008) [94]	Prospective cohort	Infant gut microbiota	Reduced early diversity associated with eczema development	Early-life microbiota influences AD risk
Nylund et al. (2014) [95]	Observational longitudinal	Gut microbiota (butyrate producers)	Higher diversity and butyrate bacteria → milder eczema	Protective role of SCFA-producing bacteria
Storrø et al. (2011) [96]	Prospective cohort	Gut microbiota	Transient microbial differences linked to IgE sensitization, not eczema	Weak association with AD
Fonseca Lahoz Melli et al. (2020) [97]	Cross-sectional	Gut microbiota	AD linked to ↑ *C. difficile*, ↓ Lactobacillus, altered composition	Distinct dysbiotic pattern in AD children
Trompette et al. (2014) [98]	Experimental (mouse model)	Gut microbiota (SCFAs)	Fiber → SCFAs → reduced allergic inflammation via GPR41	Diet-microbiota axis modulates immunity
Fang et al. (2022) [99]	Experimental + clinical	Gut microbiota – *B. longum* (I3C metabolite)	I3C activates AHR → reduces TH2 response	Improves AD symptoms via gut-skin axis
Song et al. (2016) [100]	Observational (metagenomics)	Gut microbiota – *F. prausnitzii*	Dysbiosis with reduced SCFAs and altered strains	Impaired gut barrier → promotes AD inflammation
Penders et al. (2006) [101]	Prospective cohort	Gut microbiota – *E. coli*, *C. difficile*	Early colonization increases risk of eczema and atopy	Microbiota precedes disease development
Wrześniewska et al. (2024) [102]	Review	Gut-skin axis	SCFAs regulate immunity and barrier	Probiotics/diet influence AD
Reddel et al. (2019) [103]	Observational + interventional (probiotics)	Gut microbiota	AD dysbiosis with ↓ SCFA producers; limited probiotic effect	Identifies microbial biomarkers
Park et al. (2020) [104]	Prospective cohort	Gut microbiota + SCFAs	Microbiota composition predicts transient vs persistent AD	Early microbiome influences disease course

Ta et al. (2020) [105]		Longitudinal multi-omics	Gut microbiota	Early dysbiosis (↑ *E. coli*, ↓ *B. fragilis*, ↓ SCFAs)	Alters eczema risk trajectory
Trompette et al. (2022) [106]		Experimental (mouse model)	Gut microbiota (SCFAs)	Fiber/SCFAs improve skin barrier	Reduced AD severity and sensitization
Rios-Carlos et al. (2024) [107]		Review	Gut microbiota (metabolites)	SCFAs & tryptophan metabolites regulate immunity	Therapeutic potential via microbiome modulation
Yang et al. (2024) [108]		Observational (with treatment)	Gut microbiota	Dupilumab partially restores gut microbiota and metabolism	Microbiome may contribute to treatment effects
Mashiah et al. (2021) [109]		Clinical trial (open label)	Gut microbiota (FMT)	FMT reduces SCORAD and transfers microbial strains	Potential safe/effective therapy Potential safe/effective therapy
Liu et al. (2024) [110]		Randomized controlled trial	Gut microbiota (FMT)	FMT improves EASI, reduces TH2/TH17, alters microbiota	Effective and safe therapy
Jiang et al. (2023) [111]		Experimental (mouse model)	Gut microbiota (FMT)	FMT reduces inflammation and restores microbiota	Supports therapeutic potential
Deng et al. (2023) [112]		Case report	Gut + skin microbiota	WMT improves symptoms and reduces *S. aureus*	Suggests gut-skin axis involvement
Chen et al. (2022) [113]		Systematic review (protocol)	Gut microbiota (FMT in RBD)	Evaluates efficacy/safety of FMT	Not AD-specific; aims to clarify clinical utility
Ciftci N. Et Al. (2025) [114]	U	Case-control study	Gut microbiota in urticaria patients versus controls: Blastocystis focus.	Blastocystis and urticaria alter gut microbiota composition significantly.	Blastocystis dysbiosis → may alter immune regulation in urticaria
Wang x. Et al. (2021) [118]		Cross-sectional	Gut microbiota of CSU patients vs control	Microbiota ↑/↓ specific genera → fecal/serum metabolite changes CSU	Microbiota–metabolite axis → CSU development; potential biomarkers/therapy
Song Y. Et Al. (2022) [119]		Cross-sectional and case-control	Gut microbiota in H1-antihistamine–resistant CSU patients.	H1-antihistamine–resistant patients show altered gut genera and diversity.	Microbiota changes → inflammation & antihistamine resistance
Wang D. Et Al. (2020) [120]		Cross-sectional	Gut microbiota of CSU patients.	CSU microbiota alterations affect metabolome, unsaturated fatty acids, butanoate.	Microbe–metabolite profile → may influence CSU development
Huang Y. Et al (2025) [121]		Mendelian Randomization	East Asian gut → microbiota	MR analysis revealed causal links: gut bacteria, metabolites, urticaria.	Certain microbiota components may causally influence urticaria via metabolites.
Burma NE et al. 2025) [126]	AA	Review	Cutaneous and gut dysbiosis	Summarizes associations between microbial imbalance and AA	Supports potential role of microbiome in immune dysregulation

Severino A. et al. (2025) [127]		Review	Gut microbiota	Highlights multifaceted effects of gut microbiota on	Suggests gut microbiota as a modulator of disease
Gómez-Arias PJ et al. (2024) [129]		Observational / Scalp microbiome	Scalp microbiota imbalance	Correlates microbial changes with disease severity and systemic inflammatory markers	Links local dysbiosis to severity and inflammation
Nikoloudaki O. et al. (2024) [130]		Observational / Gut microbiome + metabolome	Gut microbiome composition & metabolites	AA subjects show reduced richness and distinct taxa (Firmicutes, Lachnospirales, Blautia); metabolite biomarkers identified	Highlights gut dysbiosis and metabolic shifts; potential predictors of AA
Sánchez-Pellicer P. et al. (2022) [131]		Review	Gut microbiome	Discusses influence of microbiome on AA pathogenesis	Supports gut–skin axis as a framework for AA
Dellacecca ER et al. (2020) [137]	**V**	Experimental / Mouse model	Antibiotic induced gut dysbiosis	Antibiotic-driven microbial imbalance promotes vitiligo development	Shows causal link in mice between microbiome disruption
Bzioueche H et al. (2021) [138]		Observational / Human	Skin and gut microbiome	Matched skin–gut analysis shows deep skin dysbiosis, links to mitochondrial and immune changes	Supports microbiome alterations in human vitiligo and potential mechanistic relevance
Arpaia N et al. (2013) [139]		Experimental / Mouse model	Commensal bacterial metabolites	SCFA metabolites promote peripheral Treg generation	Provides mechanistic link between microbiota-derived metabolites and immune regulation
Smith PM et al. (2013) [140]		Experimental / Mouse model	Short-chain fatty acids (SCFAs)	SCFAs regulate colonic Treg homeostasis	Highlights microbiota metabolites shaping systemic immune tolerance
Donohoe DR et al. (2011) [141]		Experimental / Mouse model	Gut microbiome-derived butyrate	Modulates energy metabolism and autophagy	Shows microbial metabolites can affect host metabolism and autophagy pathways
Xiao X et al. (2023) [142]		Review	SCFAs	SCFAs implicated in inflammatory skin diseases	Suggests SCFA metabolites may modulate skin immune responses

References

20. Zheng, Y.; Hunt, R.L.; Villaruz, A.E.; Fisher, E.L.; Liu, R.; Liu, Q.; Cheung, G.Y.; Li, M.; Otto, M. Commensal *Staphylococcus epidermidis* contributes to skin barrier homeostasis by generating protective ceramides. *Cell Host Microbe* **2022**, *30*, 301–313.e9. https://doi.org/10.1016/j.chom.2022.01.004. PubMed PMID: 35123653; PubMed Central PMCID: PMC8917079.

38. Rendon, A.; Schäkel, K. Psoriasis Pathogenesis and Treatment. *Int. J. Mol. Sci.* **2019**, *20*, 1475. https://doi.org/10.3390/ijms20061475. PubMed PMID: 30909615.

39. Medovic, M.V.; Jakovljevic, V.L.; Zivkovic, V.I.; Jeremic, N.S.; Jeremic, J.N.; Bolevich, S.B.; Nikolic, A.B.R.; Milicic, V.M.; Srejovic, I.M. Psoriasis between Autoimmunity and Oxidative Stress: Changes Induced by Different Therapeutic Approaches. *Oxid. Med. Cell. Longev.* **2022**, *2022*, 2249834. https://doi.org/10.1155/2022/2249834. PubMed PMID: 35313642; PubMed Central PMCID: PMC8934232.

40. Lee, Y.S.; Cheon, I.S.; Kim, B.H.; Kwon, M.J.; Lee, H.W.; Kim, T.Y. Loss of Extracellular Superoxide Dismutase Induces Severe IL-23-Mediated Skin Inflammation in Mice. *J. Investig. Dermatol.* **2013**, *133*, 732–741. https://doi.org/10.1038/jid.2012.406.

41. Oszukowska, M.; Kozłowska, M.; Kaszuba, A. Paraoxonase-1 and other factors related to oxidative stress in psoriasis. *Postep. Dermatol. Alergol.* **2020**, *37*, 92–96. https://doi.org/10.5114/ada.2020.93386. PubMed PMID: 32467691; PubMed Central PMCID: PMC7247073.

42. Codoñer, F.M.; Ramírez-Bosca, A.; Climent, E.; Carrión-Gutierrez, M.; Guerrero, M.; Pérez-Orquín, J.M.; de la Parte, J.H.; Genovés, S.; Ramón, D.; Navarro-López, V.; et al. Gut microbial composition in patients with psoriasis. *Sci. Rep.* **2018**, *8*, 3812. https://doi.org/10.1038/s41598-018-22125-y. PubMed PMID: 29491401; PubMed Central PMCID: PMC5830498.

43. Ten Bergen, L.L.; Petrovic, A.; Aarebrot, A.K.; Appel, S. Current knowledge on autoantigens and autoantibodies in psoriasis. *Scand. J. Immunol.* **2020**, *92*, e12945. https://doi.org/10.1111/sji.12945. PubMed PMID: 32697368.

44. Hawkes, J.E.; Chan, T.C.; Krueger, J.G. Psoriasis pathogenesis and the development of novel targeted immune therapies. *J. Allergy Clin. Immunol.* **2017**, *140*, 645–653. https://doi.org/10.1016/j.jaci.2017.07.004. PubMed PMID: 28887948; PubMed Central PMCID: PMC5600287.

45. Sikora, M.; Stec, A.; Chrabaszcz, M.; Waskiel-Burnat, A.; Zaremba, M.; Olszewska, M.; Rudnicka, L. Intestinal Fatty Acid Binding Protein, a Biomarker of Intestinal Barrier, is Associated with Severity of Psoriasis. *J. Clin. Med.* **2019**, *8*, 1021. https://doi.org/10.3390/jcm8071021.

46. Nakai, K.; Yoneda, K.; Maeda, R.; Munehiro, A.; Fujita, N.; Yokoi, I.; Moriue, J.; Moriue, T.; Kosaka, H.; Kubota, Y. Urinary biomarker of oxidative stress in patients with psoriasis vulgaris and atopic dermatitis. *J. Eur. Acad. Dermatol. Venereol.* **2009**, *23*, 1405–1408. https://doi.org/10.1111/j.1468-3083.2009.03327.x.

47. Zákostelská, Z.; Málková, J.; Klimešová, K.; Rossmann, P.; Hornová, M.; Novosádová, I.; Stehlíková, Z.; Kostovcikova, M.; Hudcovic, T.; Štepánková, R.; et al. Intestinal Microbiota Promotes Psoriasis-Like Skin Inflammation by Enhancing Th17 Response. *PLoS ONE* **2016**, *11*, e0159539. https://doi.org/10.1371/journal.pone.0159539.

49. Shou, Y.; Yang, L.; Yang, Y.; Xu, J. Inhibition of keratinocyte ferroptosis suppresses psoriatic inflammation. *Cell Death Dis.* **2021**, *12*, 1009. https://doi.org/10.1038/s41419-021-04284-5.

52. Assarsson, M.; Duvetorp, A.; Dienus, O.; Söderman, J.; Seifert, O. Significant Changes in the Skin Microbiome in Patients with Chronic Plaque Psoriasis after Treatment with Narrowband Ultraviolet B. *Acta Derm. Venereol.* **2018**, *98*, 428–436. https://doi.org/10.2340/00015555-2859. PubMed PMID: 29199351.

53. Langan, E.A.; Künstner, A.; Miodovnik, M.; Zillikens, D.; Thaçi, D.; Baines, J.F.; Ibrahim, S.; Solbach, W.; Knobloch, J. Combined culture and metagenomic analyses reveal significant shifts in the composition of the cutaneous microbiome in psoriasis. *Br. J. Dermatol.* **2019**, *181*, 1254–1264. https://doi.org/10.1111/bjd.17989. PubMed PMID: 30985920.

55. Scaglione, G.L.; Fania, L.; De Paolis, E.; De Bonis, M.; Mazzanti, C.; Di Zenzo, G.; Lechiancole, S.; Messinese, S.; Capoluongo, E. Evaluation of cutaneous, oral and intestinal microbiota in patients affected by pemphigus and bullous pemphigoid: A pilot study. *Exp. Mol. Pathol.* **2020**, *112*, 104331. https://doi.org/10.1016/j.yexmp.2019.104331. PubMed PMID: 31705881.

56. Wang, Y.; Xia, X.; Zhou, X.; Zhan, T.; Dai, Q.; Zhang, Y.; Zhang, W.; Shu, Y.; Li, W.; Xu, H. Association of gut microbiome and metabolites with onset and treatment response of patients with pemphigus vulgaris. *Front. Immunol.* **2023**, *14*, 1114586. https://doi.org/10.3389/fimmu.2023.1114586. PubMed PMID: 37122759; PubMed Central PMCID: PMC10140300.

57. Guo, Z.; Yiu, N.; Hu, Z.; Zhou, W.; Long, X.; Yang, M.; Liao, J.; Zhang, G.; Lu, Q.; Zhao, M. Alterations of fecal microbiome and metabolome in pemphigus patients. *J. Autoimmun.* **2023**, *141*, 103108. https://doi.org/10.1016/j.jaut.2023.103108. PubMed PMID: 37714737.

58. Huang, S.; Mao, J.; Zhou, L.; Xiong, X.; Deng, Y. The imbalance of gut microbiota and its correlation with plasma inflammatory cytokines in pemphigus vulgaris patients. *Scand. J. Immunol.* **2019**, *90*, e12799. https://doi.org/10.1111/sji.12799. PubMed PMID: 31211854; PubMed Central PMCID: PMC9286422.

59. Li, S.Z.; Wu, Q.Y.; Fan, Y.; Guo, F.; Hu, X.M.; Zuo, Y.G. Gut Microbiome Dysbiosis in Patients with Pemphigus and Correlation with Pathogenic Autoantibodies. *Biomolecules* **2024**, *14*, 880. https://doi.org/10.3390/biom14070880.

60. You, S.; Ouyang, J.; Wu, Q.; Zhang, Y.; Gao, J.; Luo, X.; Wang, Y.; Wu, Y.; Jiang, F. Comparison of serum cytokines and chemokines levels and clinical significance in patients with pemphigus vulgaris-A retrospective study. *Exp. Dermatol.* **2024**, *33*, e15173. https://doi.org/10.1111/exd.15173. PubMed PMID: 39246287.

61. Yesilova, Y.; Ucmak, D.; Selek, S.; Dertlioğlu, S.B.; Sula, B.; Bozkus, F.; Turan, E. Oxidative stress index may play a key role in patients with pemphigus vulgaris. *J. Eur. Acad. Dermatol. Venereol.* **2013**, *27*, 465–467. https://doi.org/10.1111/j.1468-3083.2012.04463.x. PubMed PMID: 22324759.

62. Abida, O.; Ben Mansour, R.; Gargouri, B.; Ben Ayed, M.; Masmoudi, A.; Turki, H.; Masmoudi, H.; Lassoued, S. Catalase and lipid peroxidation values in serum of Tunisian patients with pemphigus vulgaris and foliaceus. *Biol. Trace Elem. Res.* **2012**, *150*, 74–80. https://doi.org/10.1007/s12011-012-9497-3. PubMed PMID: 22907559.

63. Huang, Y.; Jedličková, H.; Cai, Y.; Rehman, A.; Gammon, L.; Ahmad, U.S.; Uttagomol, J.; Parkinson, E.K.; Fortune, F.; Wan, H. Oxidative Stress-Mediated YAP Dysregulation Contributes to the Pathogenesis of Pemphigus Vulgaris. *Front. Immunol.* **2021**, *12*, 649502. https://doi.org/10.3389/fimmu.2021.649502.

65. Luo, Y.; Hu, J.; Zhou, Z.; Zhang, Y.; Wu, Y.; Sun, J. Oxidative stress products and managements in atopic dermatitis. *Front. Med.* **2025**, *12*, 1538194. https://doi.org/10.3389/fmed.2025.1538194.

66. Hoyer, A.; Rehbinder, E.M.; Färdig, M.; Asad, S.; Lødrup Carlsen, K.C.; Endre, K.M.A.; Granum, B.; Haugen, G.; Hedlin, G.; Jonassen, C.M.; et al. Filaggrin mutations in relation to skin barrier and atopic dermatitis in early infancy*. *Br. J. Dermatol.* **2022**, *186*, 544–552. https://doi.org/10.1111/bjd.20831.

67. Deng, L.; Costa, F.; Blake, K.J.; Choi, S.; Chandrabalan, A.; Yousuf, M.S.; Shiers, S.; Dubreuil, D.; Vega-Mendoza, D.; Rolland, C.; et al. *S. aureus* drives itch and scratch-induced skin damage through a V8 protease-PAR1 axis. *Cell* **2023**, *186*, 5375–5393.e25. https://doi.org/10.1016/j.cell.2023.10.019.

70. Furue, M. Regulation of Skin Barrier Function via Competition between AHR Axis versus IL-13/IL-4–JAK–STAT6/STAT3 Axis: Pathogenic and Therapeutic Implications in Atopic Dermatitis. *J. Clin. Med.* **2020**, *9*, 3741. https://doi.org/10.3390/jcm9113741.

71. Yan, C.; Ying, J.; Lu, W.; Changzhi, Y.; Qihong, Q.; Jingzhu, M.; Dongjie, S.; Tingting, Z. MiR-1294 suppresses ROS-dependent inflammatory response in atopic dermatitis via restraining STAT3/NF-κB pathway. *Cell. Immunol.* **2022**, *371*, 104452. https://doi.org/10.1016/j.cellimm.2021.104452.

72. Dao, T.T.P.; Song, K.; Kim, J.Y.; Kim, Y.S. Igalan from *Inula helenium* (L.) suppresses the atopic dermatitis-like response in stimulated HaCaT keratinocytes via JAK/STAT3 signaling. *Inflamm. Res.* **2020**, *69*, 309–319. https://doi.org/10.1007/s00011-020-01322-4.

73. Borgia, F.; Custurone, P.; Peterle, L.; Pioggia, G.; Gangemi, S. Role of Epithelium-Derived Cytokines in Atopic Dermatitis and Psoriasis: Evidence and Therapeutic Perspectives. *Biomolecules* **2021**, *11*, 1843. https://doi.org/10.3390/biom11121843.

74. Ayala, A.; Muñoz, M.F.; Argüelles, S. Lipid Peroxidation: Production, Metabolism, and Signaling Mechanisms of Malondialdehyde and 4-Hydroxy-2-Nonenal. *Oxidative Med. Cell. Longev.* **2014**, *2014*, 360438. https://doi.org/10.1155/2014/360438.

75. Esterbauer, H.; Schaur, R.J.; Zollner, H. Chemistry and biochemistry of 4-hydroxynonenal, malonaldehyde and related aldehydes. *Free Radic. Biol. Med.* **1991**, *11*, 81–128. https://doi.org/10.1016/0891-5849(91)90192-6.

76. Traverso, N.; Menini, S.; Maineri, E.P.; Patriarca, S.; Odetti, P.; Cottalasso, D.; Marinari, U.M.; Pronzato, M.A. Malondialdehyde, a Lipoperoxidation-Derived Aldehyde, Can Bring About Secondary Oxidative Damage To Proteins. *J. Gerontol. Ser. A: Biol. Sci. Med. Sci.* **2004**, *59*, B890–5. https://doi.org/10.1093/gerona/59.9.B890.

77. Tuma, D.J.; Kearley, M.L.; Thiele, G.M.; Worrall, S.; Haver, A.; Klassen, L.W.; Sorrell, M.F. Elucidation of Reaction Scheme Describing Malondialdehyde−Acetaldehyde−Protein Adduct Formation. *Chem. Res. Toxicol.* **2001**, *14*, 822–832. https://doi.org/10.1021/tx000222a.

79. Cheng, J.; Wang, F.; Yu, D.F.; Wu, P.F.; Chen, J.G. The cytotoxic mechanism of malondialdehyde and protective effect of carnosine via protein cross-linking/mitochondrial dysfunction/reactive oxygen species/MAPK pathway in neurons. *Eur. J. Pharmacol.* **2011**, *650*, 184–194. https://doi.org/10.1016/j.ejphar.2010.09.033.

80. Siems, W.; Grune, T. Intracellular metabolism of 4-hydroxynonenal. *Mol. Asp. Med.* **2003**, *24*, 167–75. https://doi.org/10.1016/s0098-2997(03)00011-6. PubMed PMID: 12892994.

81. Amin, M.N.; Liza, K.F.; Md Sarwar, S.; Ahmed, J.; Md Adnan, T.; Chowdhury, M.I.; Hossain, M.Z.; Islam, M.S. Effect of lipid peroxidation, antioxidants, macro minerals and trace elements on eczema. *Arch. Dermatol. Res.* **2015**, *307*, 617–623. https://doi.org/10.1007/s00403-015-1570-2.

82. Feng, Z.; Qin, Y.; Huo, F.; Jian, Z.; Li, X.; Geng, J.; Li, Y.; Wu, J. NMN recruits GSH to enhance GPX4-mediated ferroptosis defense in UV irradiation induced skin injury. *Biochim. Et. Biophys. Acta (BBA)—Mol. Basis Dis.* **2022**, *1868*, 166287. https://doi.org/10.1016/j.bbadis.2021.166287.

83. Emmert, H.; Fonfara, M.; Rodriguez, E.; Weidinger, S. NADPH oxidase inhibition rescues keratinocytes from elevated oxidative stress in a 2D atopic dermatitis and psoriasis model. *Exp. Dermatol.* **2020**, *29*, 749–758. https://doi.org/10.1111/exd.14148.

84. Kalankariyan, S.; Thottapillil, A.; Saxena, A.; Srivatsn S, M.; Kadamkode, V.; Kapoor, R.; Mitra, R.; Raut, J.; Venkatesh, K. An in silico approach deciphering the commensal dynamics in the cutaneous milieu. *npj Syst. Biol. Appl.* **2025**, *11*, 42. https://doi.org/10.1038/s41540-025-00524-y.

85. Byrd, A.L.; Deming, C.; Cassidy, S.K.B.; Harrison, O.J.; Ng, W.I.; Conlan, S.; Belkaid, Y.; Segre, J.A.; Kong, H.H.; Program, N.C.S. *Staphylococcus aureus* and *Staphylococcus epidermidis* strain diversity underlying pediatric atopic dermatitis. *Sci. Transl. Med.* **2017**, *9*, eaal4651. https://doi.org/10.1126/scitranslmed.aal4651.

86. Lai, Y.; Di Nardo, A.; Nakatsuji, T.; Leichtle, A.; Yang, Y.; Cogen, A.L.; Wu, Z.-R.; Hooper, L.V.; Schmidt, R.R.; von Aulock, S.; et al. Commensal bacteria regulate Toll-like receptor 3–dependent inflammation after skin injury. *Nat. Med.* **2009**, *15*, 1377–1382. https://doi.org/10.1038/nm.2062.

87. Paller, A.S.; Kong, H.H.; Seed, P.; Naik, S.; Scharschmidt, T.C.; Gallo, R.L.; Luger, T.; Irvine, A.D. The microbiome in patients with atopic dermatitis. *J. Allergy Clin. Immunol.* **2019**, *143*, 26–35. https://doi.org/10.1016/j.jaci.2018.11.015.

88. Cau, L.; Williams, M.R.; Butcher, A.M.; Nakatsuji, T.; Kavanaugh, J.S.; Cheng, J.Y.; Shafiq, F.; Higbee, K.; Hata, T.R.; Horswill, A.R.; et al. *Staphylococcus epidermidis* protease EcpA can be a deleterious component of the skin microbiome in atopic dermatitis. *J. Allergy Clin. Immunol.* **2021**, *147*, 955–966.e16. https://doi.org/10.1016/j.jaci.2020.06.024.

89. Scharschmidt, T.C.; Fischbach, M.A. What lives on our skin: Ecology, genomics and therapeutic opportunities of the skin microbiome. *Drug Discov. Today Dis. Mech.* **2013**, *10*, e83–9. https://doi.org/10.1016/j.ddmec.2012.12.003.

90. Almoughrabie, S.; Cau, L.; Cavagnero, K.; O'Neill, A.M.; Li, F.; Roso-Mares, A.; Mainzer, C.; Closs, B.; Kolar, M.J.; Williams, K.J.; et al. Commensal *Cutibacterium acnes* induce epidermal lipid synthesis important for skin barrier function. *Sci. Adv.* **2023**, *9*, eadg6262. https://doi.org/10.1126/sciadv.adg6262.

91. Tudela, H.; Claus, S.P.; Saleh, M. Next Generation Microbiome Research: Identification of Keystone Species in the Metabolic Regulation of Host-Gut Microbiota Interplay. *Front. Cell Dev. Biol.* **2021**, *9*, 719072. https://doi.org/10.3389/fcell.2021.719072.

92. Alam, M.J.; Xie, L.; Yap, Y.A.; Marques, F.Z.; Robert, R. Manipulating Microbiota to Treat Atopic Dermatitis: Functions and Therapies. *Pathogens* **2022**, *11*, 642. https://doi.org/10.3390/pathogens11060642.

93. Malgesini, A.; Marsiglia, M.D.; Borghi, E.; Marzano, A.V.; Nazzaro, G. The Emerging Role of Gut Microbiota in Inflammatory Skin Diseases: A Systematic Review. *Exp. Dermatol.* **2026**, *35*, e70234. https://doi.org/10.1111/exd.70234.

94. Wang, M.; Karlsson, C.; Olsson, C.; Adlerberth, I.; Wold, A.E.; Strachan, D.P.; Martricardi, P.M.; Åberg, N.; Perkin, M.R.; Tripodi, S.; et al. Reduced diversity in the early fecal microbiota of infants with atopic eczema. *J. Allergy Clin. Immunol.* **2008**, *121*, 129–134. https://doi.org/10.1016/j.jaci.2007.09.011.

95. Nylund, L.; Nermes, M.; Isolauri, E.; Salminen, S.; De Vos, W.M.; Satokari, R. Severity of atopic disease inversely correlates with intestinal microbiota diversity and butyrate-producing bacteria. *Allergy* **2015**, *70*, 241–244. https://doi.org/10.1111/all.12549.

96. Storrø, O.; Øien, T.; Langsrud, Ø.; Rudi, K.; Dotterud, C.; Johnsen, R. Temporal variations in early gut microbial colonization are associated with allergen-specific immunoglobulin E but not atopic eczema at 2 years of age. *Clin. Exp. Allergy* **2011**, *41*, 1545–1554. https://doi.org/10.1111/j.1365-2222.2011.03817.x.

97. Fonseca Lahoz Melli, L.C.; Carmo-Rodrigues, M.S.D.; Araújo-Filho, H.B.; Santos Mello, C.; Tahan, S.; Campos Pignatari, A.C.; Solé, D.; De Morais, M.B. Gut microbiota of children with atopic dermatitis: Controlled study in the metropolitan region of São Paulo, Brazil. *Allergol. Immunopathol.* **2020**, *48*, 107–115. https://doi.org/10.1016/j.aller.2019.08.004.

98. Trompette, A.; Gollwitzer, E.S.; Yadava, K.; Sichelstiel, A.K.; Sprenger, N.; Ngom-Bru, C.; Blanchard, C.; Junt, T.; Nicod, L.P.; Harris, N.L.; et al. Gut microbiota metabolism of dietary fiber influences allergic airway disease and hematopoiesis. *Nat. Med.* **2014**, *20*, 159–166. https://doi.org/10.1038/nm.3444.

99. Fang, Z.; Pan, T.; Li, L.; Wang, H.; Zhu, J.; Zhang, H.; Zhao, J.; Chen, W.; Lu, W. *Bifidobacterium longum* mediated tryptophan metabolism to improve atopic dermatitis via the gut-skin axis. *Gut Microbes* **2022**, *14*, 2044723. https://doi.org/10.1080/19490976.2022.2044723.

100. Song, H.; Yoo, Y.; Hwang, J.; Na, Y.C.; Kim, H.S. Faecalibacterium prausnitzii subspecies–level dysbiosis in the human gut microbiome underlying atopic dermatitis. *J. Allergy Clin. Immunol.* **2016**, *137*, 852–860. https://doi.org/10.1016/j.jaci.2015.08.021.

101. Penders, J.; Thijs, C.; Van Den Brandt, P.A.; Kummeling, I.; Snijders, B.; Stelma, F.; Adams, H.; van Ree, R.; Stobberingh, E.E. Gut microbiota composition and development of atopic manifestations in infancy: The KOALA Birth Cohort Study. *Gut* **2007**, *56*, 661–667. https://doi.org/10.1136/gut.2006.100164.

102. Wrześniewska, M.; Wołoszczak, J.; Świrkosz, G.; Szyller, H.; Gomułka, K. The Role of the Microbiota in the Pathogenesis and Treatment of Atopic Dermatitis—A Literature Review. *IJMS* **2024**, *25*, 6539. https://doi.org/10.3390/ijms25126539.

103. Reddel, S.; Del Chierico, F.; Quagliariello, A.; Giancristoforo, S.; Vernocchi, P.; Russo, A.; Fiocchi, A.; Rossi, P.; Putignani, L.; El Hachem, M. Gut microbiota profile in children affected by atopic dermatitis and evaluation of intestinal persistence of a probiotic mixture. *Sci. Rep.* **2019**, *9*, 4996. https://doi.org/10.1038/s41598-019-41149-6.

104. Park, Y.M.; Lee, S.Y.; Kang, M.J.; Kim, B.S.; Lee, M.J.; Jung, S.S.; Yoon, J.S.; Cho, H.-J.; Lee, E.; Yang, S.-I.; et al. Imbalance of Gut *Streptococcus* , *Clostridium* , and *Akkermansia* Determines the Natural Course of Atopic Dermatitis in Infant. *Allergy Asthma Immunol. Res.* **2020**, *12*, 322. https://doi.org/10.4168/aair.2020.12.2.322.

105. Ta, L.D.H.; Chan, J.C.Y.; Yap, G.C.; Purbojati, R.W.; Drautz-Moses, D.I.; Koh, Y.M.; Tay, C.J.X.; Huang, C.-H.; Kioh, D.Y.Q.; Woon, J.Y.; et al. A compromised developmental trajectory of the infant gut microbiome and metabolome in atopic eczema. *Gut Microbes* **2020**, *12*, 1801964. https://doi.org/10.1080/19490976.2020.1801964.

106. Trompette, A.; Pernot, J.; Perdijk, O.; Alqahtani, R.A.A.; Domingo, J.S.; Camacho-Muñoz, D.; Wong, N.C.; Kendall, A.C.; Wiederkehr, A.; Nicod, L.P.; et al. Gut-derived short-chain fatty acids modulate skin barrier integrity by promoting keratinocyte metabolism and differentiation. *Mucosal Immunol.* **2022**, *15*, 908–926. https://doi.org/10.1038/s41385-022-00524-9.

107. Rios-Carlos, M.; Cervantes-García, D.; Córdova-Dávalos, L.E.; Bermúdez-Humarán, L.G.; Salinas, E. Unraveling the gut-skin axis in atopic dermatitis: Exploiting insights for therapeutic strategies. *Gut Microbes* **2024**, *16*, 2430420. https://doi.org/10.1080/19490976.2024.2430420.

108. Yang, L.; Li, D.; Sun, S.; Liu, D.; Wang, Y.; Liu, X.; Zhou, B.; Nie, W.; Li, L.; Wang, Y.; et al. Dupilumab therapy improves gut microbiome dysbiosis and tryptophan metabolism in Chinese patients with atopic dermatitis. *Int. Immunopharmacol.* **2024**, *131*, 111867. https://doi.org/10.1016/j.intimp.2024.111867.

109. Mashiah, J.; Karady, T.; Fliss-Isakov, N.; Sprecher, E.; Slodownik, D.; Artzi, O.; Samuelov, L.; Ellenbogen, E.; Godneva, A.; Segal, E.; et al. Clinical efficacy of fecal microbial transplantation treatment in adults with moderate-to-severe atopic dermatitis. *Immun. Inflam. Dis.* **2022**, *10*, e570. https://doi.org/10.1002/iid3.570.

110. Liu, X.; Luo, Y.; Chen, X.; Wu, M.; Xu, X.; Tian, J.; Gao, Y.; Zhu, J.; Wang, Z.; Zhou, Y.; et al. Fecal microbiota transplantation against moderate-to-severe atopic dermatitis: A randomized, double-blind controlled explorer trial. *Allergy* **2025**, *80*, 1377–1388. https://doi.org/10.1111/all.16372.

111. Jiang, X.; Liu, Z.; Ma, Y.; Miao, L.; Zhao, K.; Wang, D.; Wang, M.; Ruan, H.; Xu, F.; Zhou, Q.; et al. Fecal microbiota transplantation affects the recovery of AD-skin lesions and enhances gut microbiota homeostasis. *Int. Immunopharmacol.* **2023**, *118*, 110005. https://doi.org/10.1016/j.intimp.2023.110005.

112. Deng, W.Y.; Chen, W.J.; Zhong, H.J.; Wu, L.H.; He, X.X. Washed microbiota transplantation: A case report of clinical success with skin and gut microbiota improvement in an adolescent boy with atopic dermatitis. *Front. Immunol.* **2023**, *14*, 1275427. https://doi.org/10.3389/fimmu.2023.1275427.

113. Chen, Q.; Zhang, Z.; Bei, S.; Wang, X.; Zhu, Y. Efficacy of oral fecal microbiota transplantation in recurrent bowel disease: A protocol for systematic review and meta-analysis. *Medicine* **2022**, *101*, e31477. https://doi.org/10.1097/MD.0000000000031477.

114. Ciftci, N.; Macin, S.; Saylam Kurtipek, G.; Arslan, U. Comparison of the Intestinal Microbiota of Patients with Urticaria and Healthy Controls: The Role of Blastocystis. *Pathogens* **2025**, *14*, 1140. https://doi.org/10.3390/pathogens14111140.

115. Rajappa, M.; Chandrashekar, L.; Sundar, I.; Munisamy, M.; Ananthanarayanan, P.H.; Thappa, D.M.; Toi, P.C. Platelet oxidative stress and systemic inflammation in chronic spontaneous urticaria. *Clin. Chem. Lab. Med.* **2013**, *51*, 1789–1794. https://doi.org/10.1515/cclm-2012-0897.

116. Sagdic, A.; Sener, O.; Bulucu, F.; Karadurmus, N.; Yamanel, L.; Tasci, C.; Naharci, I.; Ocal, R.; Aydin, A. Oxidative stress status in patients with chronic idiopathic urticaria. *Allergol. Immunopathol.* **2011**, *39*, 150–153. https://doi.org/10.1016/j.aller.2010.06.012.

117. Raho, G.; Cassano, N.; D'Argento, V.; Vena, G.A.; Zanotti, F. Over-expression of Mn-superoxide dismutase as a marker of oxidative stress in lesional skin of chronic idiopathic urticaria: Mn-SOD as a marker in CIU. *Clin. Exp. Dermatol.* **2003**, *28*, 318–320. https://doi.org/10.1046/j.1365-2230.2003.01264.x.

118. Wang, X.; Yi, W.; He, L.; Luo, S.; Wang, J.; Jiang, L.; Long, H.; Zhao, M.; Lu, Q. Abnormalities in Gut Microbiota and Metabolism in Patients With Chronic Spontaneous Urticaria. *Front. Immunol.* **2021**, *12*, 691304. https://doi.org/10.3389/fimmu.2021.691304.

119. Song, Y.; Dan, K.; Yao, Z.; Yang, X.; Chen, B.; Hao, F. Altered Gut Microbiota in H1-Antihistamine-Resistant Chronic Spontaneous Urticaria Associates With Systemic Inflammation. *Front. Cell Infect. Microbiol.* **2022**, *12*, 831489. https://doi.org/10.3389/fcimb.2022.831489.

120. Wang, D.; Guo, S.; He, H.; Gong, L.; Cui, H. Gut Microbiome and Serum Metabolome Analyses Identify Unsaturated Fatty Acids and Butanoate Metabolism Induced by Gut Microbiota in Patients With Chronic Spontaneous Urticaria. *Front. Cell. Infect. Microbiol.* **2020**, *10*, 24. https://doi.org/10.3389/fcimb.2020.00024.

121. Huang, Y. Causal relationships among gut microbiota, blood metabolites, and urticaria in East Asians: A Mendelian randomization study. *Zhong Nan Da Xue Xue Bao Yi Xue Ban* **2025**, *50*, 1590. https://doi.org/10.11817/j.issn.1672-7347.2025.250192.

124. Peterle, L.; Sanfilippo, S.; Borgia, F.; Cicero, N.; Gangemi, S. Alopecia Areata: A Review of the Role of Oxidative Stress, Possible Biomarkers, and Potential Novel Therapeutic Approaches. *Antioxidants* **2023**, *12*, 135. https://doi.org/10.3390/antiox12010135. PubMed PMID: 36670997; PubMed Central PMCID: PMC9854963.

125. Shakoei, S.; Mirmiranpoor, H.; Nakhjavani, M.; Nasimi, M.; Bakhshi, G.; Azizpour, A. Oxidative stress and antioxidant markers in patients with alopecia areata: A comparative cross-sectional study. *Indian. J. Dermatol. Venereol. Leprol.* **2023**, *89*, 411–415. https://doi.org/10.25259/IJDVL_228_20. PubMed PMID: 35962507.

126. Burma, N.E.; Ramien, M.L. Cutaneous and Gut Dysbiosis in Alopecia Areata: A Review. *JID Innov.* **2025**, *5*, 100363. https://doi.org/10.1016/j.xjidi.2025.100363.

127. Severino, A.; Porcari, S.; Rondinella, D.; Capuano, E.; Rozera, T.; Kaitsas, F.; Gasbarrini, A.; Cammarota, G.; Ianiro, G. The Multi-Faceted Role of Gut Microbiota in Alopecia Areata. *Biomedicines* **2025**, *13*, 1379. https://doi.org/10.3390/biomedicines13061379.

129. Gómez-Arias, P.J.; Gay-Mimbrera, J.; Rivera-Ruiz, I.; Aguilar-Luque, M.; Juan-Cencerrado, M.; Mochón-Jiménez, C.; Gómez-García, F.; Sánchez-González, S.; Ortega-Hernández, A.; Gómez-Garre, D.; et al. Association Between Scalp Microbiota Imbalance, Disease Severity, and Systemic Inflammatory Markers in Alopecia Areata. *Dermatol. Ther.* **2024**, *14*, 2971–2986. https://doi.org/10.1007/s13555-024-01281-2.

130. Nikoloudaki, O.; Pinto, D.; Acin Albiac, M.; Celano, G.; Da Ros, A.; De Angelis, M.; Rinaldi, F.; Gobbetti, M.; Di Cagno, R. Exploring the Gut Microbiome and Metabolome in Individuals with Alopecia Areata Disease. *Nutrients* **2024**, *16*, 858. https://doi.org/10.3390/nu16060858.

131. Sánchez-Pellicer, P.; Navarro-Moratalla, L.; Núñez-Delegido, E.; Agüera-Santos, J.; Navarro-López, V. How Our Microbiome Influences the Pathogenesis of Alopecia Areata. *Genes* **2022**, *13*, 1860. https://doi.org/10.3390/genes13101860. PubMed PMID: 36292745; PubMed Central PMCID: PMC9601531.

135. Xie, H.; Zhou, F.; Liu, L.; Zhu, G.; Li, Q.; Li, C.; Gao, T. Vitiligo: How do oxidative stress-induced autoantigens trigger autoimmunity? *J. Dermatol. Sci.* **2016**, *81*, 3–9. https://doi.org/10.1016/j.jdermsci.2015.09.003.

136. Lee, E.J.; Kim, J.Y.; Yeo, J.H.; Park, S.; Bae, Y.J.; Kwon, I.J.; Seong, S.H.; Lee, J.; Oh, S.H. ISG15–USP18 Dysregulation by Oxidative Stress Promotes IFN-γ Secretion from CD8+ T Cells in Vitiligo. *J. Investig. Dermatol.* **2024**, *144*, 273–283.e11. https://doi.org/10.1016/j.jid.2023.08.006.

137. Dellacecca, E.R.; Cosgrove, C.; Mukhatayev, Z.; Akhtar, S.; Engelhard, V.H.; Rademaker, A.W.; Knight, K.L.; Le Poole, I.C. Antibiotics Drive Microbial Imbalance and Vitiligo Development in Mice. *J. Investig. Dermatol.* **2020**, *140*, 676–687.e6. https://doi.org/10.1016/j.jid.2019.08.435.
138. Bzioueche, H.; Simonyté Sjödin, K.; West, C.E.; Khemis, A.; Rocchi, S.; Passeron, T.; Tulic, M.K. Analysis of Matched Skin and Gut Microbiome of Patients with Vitiligo Reveals Deep Skin Dysbiosis: Link with Mitochondrial and Immune Changes. *J. Investig. Dermatol.* **2021**, *141*, 2280–2290. https://doi.org/10.1016/j.jid.2021.01.036.
139. Arpaia, N.; Campbell, C.; Fan, X.; Dikiy, S.; Van Der Veeken, J.; de Roos, P.; Liu, H.; Cross, J.R.; Pfeffer, K.; Coffer, P.J.; et al. Metabolites produced by commensal bacteria promote peripheral regulatory T-cell generation. *Nature* **2013**, *504*, 451–455. https://doi.org/10.1038/nature12726.
140. Smith, P.M.; Howitt, M.R.; Panikov, N.; Michaud, M.; Gallini, C.A.; Bohlooly-Y, M.; Glickman, J.N.; Garrett, W.S. The Microbial Metabolites, Short-Chain Fatty Acids, Regulate Colonic T_{reg} Cell Homeostasis. *Science* **2013**, *341*, 569–573. https://doi.org/10.1126/science.1241165.
141. Donohoe, D.R.; Garge, N.; Zhang, X.; Sun, W.; O'Connell, T.M.; Bunger, M.K.; Bultman, S.J. The Microbiome and Butyrate Regulate Energy Metabolism and Autophagy in the Mammalian Colon. *Cell Metab.* **2011**, *13*, 517–526. https://doi.org/10.1016/j.cmet.2011.02.018.
142. Xiao, X.; Hu, X.; Yao, J.; Cao, W.; Zou, Z.; Wang, L.; Qin, H.; Zhong, D.; Li, Y.; Xue, P.; et al. The role of short-chain fatty acids in inflammatory skin diseases. *Front. Microbiol.* **2023**, *13*, 1083432. https://doi.org/10.3389/fmicb.2022.1083432.
